# Evolution of the Tetrapyrrole Biosynthetic Pathway in Secondary Algae: Conservation, Redundancy and Replacement

**DOI:** 10.1371/journal.pone.0166338

**Published:** 2016-11-18

**Authors:** Jaromír Cihlář, Zoltán Füssy, Aleš Horák, Miroslav Oborník

**Affiliations:** 1 Biology Centre, Czech Academy of Sciences, Institute of Parasitology, České Budějovice, Czech Republic; 2 University of South Bohemia, Faculty of Science, České Budějovice, Czech Republic; 3 Institute of Microbiology, Czech Academy of Sciences, Třeboň, Czech Republic; Institut de Genetique et Developpement de Rennes, FRANCE

## Abstract

Tetrapyrroles such as chlorophyll and heme are indispensable for life because they are involved in energy fixation and consumption, i.e. photosynthesis and oxidative phosphorylation. In eukaryotes, the tetrapyrrole biosynthetic pathway is shaped by past endosymbioses. We investigated the origins and predicted locations of the enzymes of the heme pathway in the chlorarachniophyte *Bigelowiella natans*, the cryptophyte *Guillardia theta*, the “green” dinoflagellate *Lepidodinium chlorophorum*, and three dinoflagellates with diatom endosymbionts (“dinotoms”): *Durinskia baltica*, *Glenodinium foliaceum* and *Kryptoperidinium foliaceum*. *Bigelowiella natans* appears to contain two separate heme pathways analogous to those found in *Euglena gracilis*; one is predicted to be mitochondrial-cytosolic, while the second is predicted to be plastid-located. In the remaining algae, only plastid-type tetrapyrrole synthesis is present, with a single remnant of the mitochondrial-cytosolic pathway, a ferrochelatase of *G*. *theta* putatively located in the mitochondrion. The green dinoflagellate contains a single pathway composed of mostly rhodophyte-origin enzymes, and the dinotoms hold two heme pathways of apparently plastidal origin. We suggest that heme pathway enzymes in *B*. *natans* and *L*. *chlorophorum* share a predominantly rhodophytic origin. This implies the ancient presence of a rhodophyte-derived plastid in the chlorarachniophyte alga, analogous to the green dinoflagellate, or an exceptionally massive horizontal gene transfer.

## Introduction

Plastid acquisitions are rare evolutionary events that give host cells the metabolic capacities of their new photosynthetic organelles. While there are only two documented primary plastid acquisitions [1,2], involving a eukaryote as host and cyanobacterium as the endosymbiont, the history of higher order eukaryote-to-eukaryote acquisitions is intensely debated [3–8]. Based on available data, it is believed that the red plastids of cryptophytes, alveolates, stramenopiles, and haptophytes (the “CASH taxa”) originate from a single ancient event with a rhodophyte alga as the endosymbiont [9–14]. However, phylogenies of the host organisms are often incompatible with this scenario [6,15–18], suggesting that the plastid was transferred horizontally in at least some of these lineages. Furthermore, higher order endosymbioses and horizontal gene transfer (HGT) may be blurring our vision of eukaryotic evolution [7]. For instance, some dinoflagellates replaced their ancestral peridinin-pigmented plastids with plastids originating from serial secondary or tertiary endosymbioses [7,19–23]. The original red plastid was replaced by a secondary green plastid in *Lepidodinium chlorophorum* [24,25]; while in so-called dinotoms (*Glenodinium foliaceum*, *Durinskia baltica*), the newly obtained tertiary endosymbiont is an engulfed diatom [26,27]. Serial plastid endosymbioses are sometimes discernible by phylogenetic signal (e.g. if a green plastid replaces a red one). However, ancient events can still be difficult to pinpoint, which might account for the contradictory and peculiar phylogenetic signals observed—for example, the number of green genes in the CASH taxa [28–33] and the proposed independent origin of plastid genes in two main branches of alveolates, the dinoflagellates and apicomplexans [34,35]. In contrast, chlorarachniophytes and phototrophic euglenids acquired green plastids and their extant relatives are heterotrophic, allowing for straight-forward evolutionary interpretations of gene origins, based on phylogenetic clustering with their heterotrophic kin or with the chlorophyte plastid donors [31,36,37].

The process of endosymbiosis involves endosymbiont genome reduction via gene transfer to the host nucleus [38,39], allowing for enhanced host control over the organelle [40,41] and reduced functional redundancy of cellular biochemistry [42]. However, the level of reduction differs among algae possessing complex photosynthetic organelles. Most of them, such as diatoms, dinoflagellates or phototrophic euglenids, have a highly reduced algal endosymbiont with multiple membranes surrounding the plastid as the only apparent morphological footprints revealing past complex endosymbioses. Organelle reduction tends to be higher in cases of older symbioses, but also depends on other factors including plastid number and evolutionary constraints [43,44]. For instance, cryptophyte and chlorarachniophyte plastids seem to be evolutionarily frozen and retain a remnant nucleus (nucleomorph) that provides genetic material required, e.g. for the maintenance of protein import mechanisms [31,45,46]. In dinotoms, the diatom endosymbiont still contains a plastid, a mitochondrion and a nucleus and is thought to represent an almost entirely independent cellular compartment [47,48]. Furthermore, it appears that the host dinotom cell holds a metabolically active remnant of the original peridinin-pigmented plastid, presumably the eyespot [47,49].

**Fig 1:**
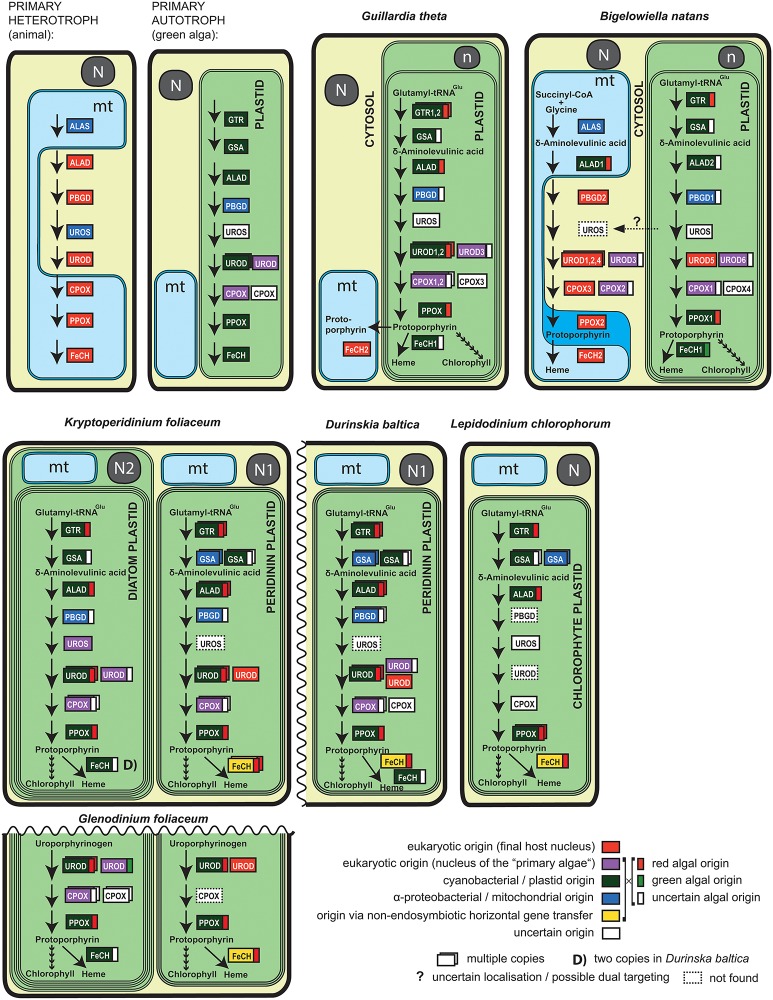
Arrangement of the heme biosynthetic pathway in algae with complex plastids. Inferred origins of enzymes are represented by colored boxes with flags where applicable. Localizations of *B*. *natans* and *G*. *theta* enzymes were predicted by SignalP and TargetP (see Material and Methods). Dashed arrows indicate a possible dual localization of UROS in both the cytosol and the plastid of *B*. *natans*. Only key metabolites are shown for clarity, for example the starting substrates for mitochondrial-cytosolic C4 (succinyl-CoA and glycine) and plastid C5 pathways (glutamyl-tRNA^Glu^). Parts of the pathway identical to *K*. *foliaceum* are not depicted in the *D*. *baltica* and *G*. *foliaceum* scheme. Schematic representation of organelles: N, N1 –nucleus of the host; N2 –nucleus of the endosymbiont diatom; n—nucleomorph of the endosymbiont; mt—mitochondrion. Enzymes: ALAS—delta-aminolevulinic acid synthase; GTR—glutamate-tRNA reductase; GSA—glutamate-1-semialdehyde 2,1-aminotransferase; ALAD—aminolevulinic acid dehydratase; PBGD—porphobilinogen deaminase; UROS—uroporphyrinogen-III synthase; UROD—uroporphyrinogen decarboxylase; CPOX—coproporphyrinogen oxidase; PPOX—protoporphyrinogen oxidase; FeCH—ferrochelatase. A typical pathway in a primary heterotroph and a primary autotroph are shown for comparison (Kořený and Oborník 2011).

One of the essential biochemical pathways carried out in plastids is tetrapyrrole synthesis. Tetrapyrroles are cyclic porphyrins coordinated by iron (heme) or magnesium (chlorophyll). They are essential for life, since heme is a substantial component of the respiratory chain and chlorophyll is an indispensable compound in the conversion of light energy to chemical bonds in carbohydrates through photosynthesis. Although the kinetoplastid flagellate *Phytomonas serpens* has been shown to be able to live in the absence of heme, it is an extremely rare metabolic deviation [50]. In phototrophic eukaryotes, tetrapyrroles are required in three cellular compartments: the cytosol, the mitochondrion, and the plastid. Most phototrophs synthesize tetrapyrrole compounds exclusively in the plastid and transport them to other compartments (overview in Fig 1). The biosynthetic process, however, can be more complex; in the excavate alga *Euglena gracilis* two independent tetrapyrrole biosynthesis pathways are present [51–55], likely because of the recent acquisition of the secondary green plastid [36,37]. These parallel tetrapyrrole pathways differ in both evolutionary origin and starting substrate. One pathway derives from the heterotrophic (secondary) host and uses condensation of succinyl-CoA and glycine (via the C4 pathway) to synthesize δ-aminolevulinic acid (ALA), the first common precursor, in the mitochondrion. The heterotrophic-type synthesis takes place partly in the mitochondrion and partly in the cytosol as it does in eukaryotic primary heterotrophs [55,56]. The other pathway is located entirely in the plastid and generates ALA from glutamate (via the C5 pathway). Evolutionarily, the plastid pathway originates from the green algal endosymbiont and, ultimately, from the cyanobacterium engulfed during primary endosymbiosis. In *E*. *gracilis*, the two tetrapyrrole pathways do not overlap, and thus produce tetrapyrroles separately for the cytosol and mitochondrion and for the plastid [51,54]. The presence of two redundant tetrapyrrole pathways is interpreted as an intermediate state in endosymbiosis [55] and would allow for the loss of one of the pathways in order to streamline cellular biochemistry. Usually, the mitochondrial-cytosolic pathway is lost in the course of evolution in eukaryotic phototrophs [55,57] and it is rare to see the plastid pathway disappear in exchange for the cytosolic counterpart, but the parasite *Perkinsus marinus* [58], for example, retained the heterotrophic heme synthesis pathway despite the continued presence of a relict plastid (reviewed in [59]). The alveolate alga *Chromera velia*, on the other hand, employs a hybrid tetrapyrrole pathway. Synthesis is initiated in the mitochondrion via the C4 pathway and is predicted to continue in the plastid. This hybrid synthesis qualifies *Chromera* as the only known phototroph able to synthesize tetrapyrroles from glycine [57], similar to heme biosynthetic processes in apicomplexan parasites that still possess a remnant, non-photosynthetic plastid [60].

The organization of heme synthesis is currently uncharacterized in most algae with complex plastids. In order to map the level of pathway conservation, reduction or replacement in further phototrophic lineages, we investigated phylogenetic relationships and predicted the cellular locations of enzymes involved in tetrapyrrole biosynthesis in the cryptophyte *Guillardia theta*, the chlorarachniophyte *Bigelowiella natans*, the green dinoflagellate *Lepidodinium chlorophorum* and the dinotoms *Durinskia baltica*, *Glenodinium foliaceum* and *Kryptoperidinium foliaceum*.

## Results and Discussion

### *Bigelowiella natans* possesses two heme pathways

**Fig 2:**
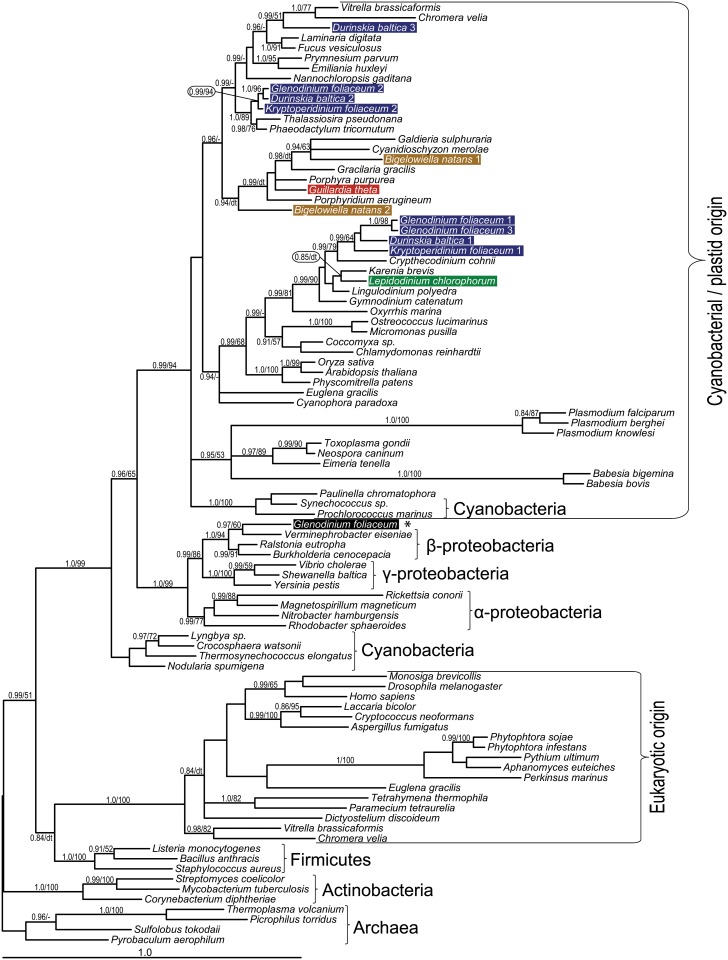
Bayesian phylogenetic tree as inferred from ALAD amino acid sequences. Taxa of interest in this study are highlighted by colored bars: blue for dinotoms, green for *Lepidodinium chlorophorum*, ochre for *Bigelowiella natans* and red for *Guillardia theta*. The tree shows red algal origin for *B*. *natans* and *G*. *theta* enzymes. For *L*. *chlorophorum*, we suggest a gene duplication / loss of paralogs scenario (see text); despite branching as sister to green algae, other dinoflagellates contained in the same clade do not possess a green algal plastid. Numbers near branches indicate Bayesian posterior probabilities followed by the bootstrap of respective clades from the likelihood analysis. Only support values greater than 0.85 (Bayesian) and 50 (likelihood) are shown. dt—different topology in the ML tree, see S2 Fig; a dash indicates an unsupported topology. An asterisk marks inferred bacterial contamination in *G*. *foliaceum* data.

In *B*. *natans*, we identified thirteen and nine sequences, respectively, of enzymes belonging to the algal endosymbiont heme pathway (autotrophic pathway) and to the heterotrophic (mitochondrial-cytosolic) pathway. The chlorarachniophyte host pathway is typical for eukaryotic heterotrophs, with ALA being synthesized by the mitochondrial C4 pathway; the enzymes involved are predicted to localize to the mitochondrion (aminolevulinic acid synthase, or BnALAS; protoporphyrinogen oxidase, BnPPOX2; and ferrochelatase, BnFeCH2) and cytosol (porphobilinogen deaminase, BnPBGD2; uroporphyrinogen decarboxylase BnUROD1-4, and coproporphyrinogen oxidase, BnCPOX2, -3) (see Fig 1, Material and Methods and S1 Table for details). An N-terminal mitochondrial transit peptide was not predicted in BnALAS, but mitochondrial transit peptides have not been detected in any eukaryotic ALAS [57] examined so far, in spite of the fact that an N-terminal extension is apparent when the eukaryotic protein is aligned to bacterial homologs (not shown) and its amino acid composition resembles that of mitochondrial transit peptides. Moreover, ALAS has never been experimentally found outside of the mitochondrion, likely because it uses succinyl-CoA, a product of the mitochondrial citrate cycle, as the initial substrate [57,61]. The host gene for aminolevulinate dehydratase (ALAD) was lost and was likely replaced by a cyanobacterial (plastid) homolog (Fig 2); a predicted mitochondrial transit peptide at its N-terminus further supports a mitochondrial location (see S1 Table for details). BnPBGD2, responsible for the next step of the mitochondrial-cytosolic pathway, forms an unsupported but stable clade with other eukaryotic sequences, branching as sister to *E*. *gracilis* and oomycetes (non-photosynthetic stramenopiles). This clade, composed of animal, fungal, (phototrophic) excavate, heterotrophic stramenopile and chlorarachniophyte sequences, very likely represents the only PBGD enzymes originating in the eukaryotic nucleus because all phototrophic eukaryotes utilize PBGD of α-proteobacterial origin (S1 Fig) [50,56,57].

In spite of the presence of two tetrapyrrole pathways in the chlorarachniophyte, only a single gene coding for a putatively plastid-targeted uroporphyrinogen-III synthase (UROS) was found in the genome database. The enzyme possesses a bipartite targeting sequence at the N-terminus, necessary for delivering the protein into the secondary plastid (Fig 1, S1 Table). Notably, all UROS genes in eukaryotic phototrophs form a single compact cluster, although the origin of this phototrophic clade is not clear (S1 Fig). Since we failed to find the cytosolic (heterotrophic) UROS in the genome, we can speculate that it was either not covered by the sequencing or annotation methods used or the recovered *B*. *natans* protein is dual-targeted to the cytosol and the plastid.

Most of the enzymes involved in the mitochondrial-cytosolic pathway are encoded by a single gene in *B*. *natans*, with the exception of UROD. All six genes coding for UROD display eukaryotic origin in *B*. *natans*; however, four of the UROD sequences (BnUROD1, -2, -4 and -5) are obvious multiple paralogs likely originating from the secondary host nucleus, while two paralogs originate from the endosymbiont (algal) nucleus (BnUROD3 and -6) (Fig 1 and S1 Fig). According to predictions, three UROD enzymes (BnUROD1, -2, -3) are cytosolic, while three other URODs appear to be plastid-located, and heterotrophic enzyme BnUROD5 may have functionally replaced the cyanobacterial counterparts in the autotrophic pathway (S1 Table).

**Fig 3:**
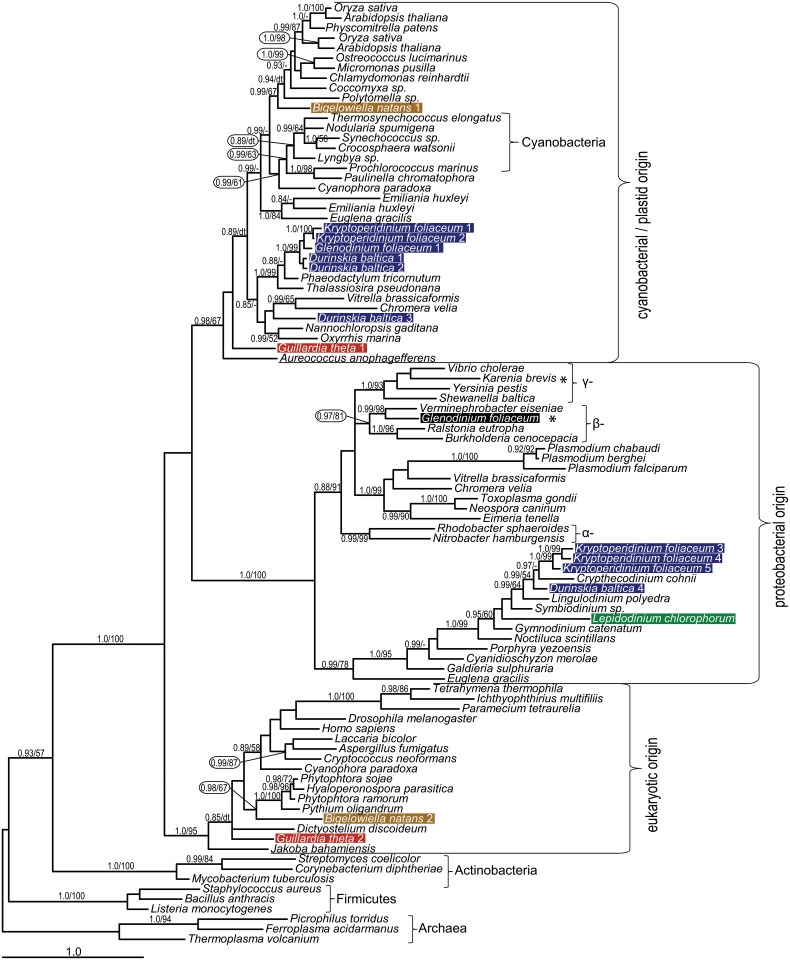
Bayesian phylogenetic tree as inferred from FeCH amino acid sequences. Taxa of interest in this study are highlighted by colored bars: blue for dinotoms, green for *Lepidodinium chlorophorum*, ochre for *Bigelowiella natans* and red for *Guillardia theta*. We document two orthologs, one of unresolved cyanobacterial origin and the other of eukaryotic origin, for *B*. *natans* and *G*. *theta* enzymes. The *L*. *chlorophorum* sequence branches together with other dinoflagellates, suggesting its origin lies in the peridinin plastid repertoire. Numbers near branches indicate Bayesian posterior probabilities followed by the bootstrap of respective clades from the likelihood analysis. Only support values greater than 0.85 (Bayesian) and 50 (likelihood) are shown. dt—different topology in the ML tree, see S2 Fig; a dash indicates unsupported topology. An asterisk marks inferred bacterial contamination in *G*. *foliaceum* and *Karenia brevis* data.

There are two genes coding for CPOX (BnCPOX2, -3) predicted to function within the heterotrophic pathway in *B*. *natans*; the former appeared within the clade composed of red-derived secondary algae, and the latter has a nuclear origin with an unsupported sister position to ciliate sequences (S1 Fig). A eukaryotic origin is also suggested for the putatively mitochondrion-located BnPPOX2 in *B*. *natans*. The eukaryotic clade is supported by Bayesian analysis in this case but its internal structure is not resolved, forming numerous polytomies (S1 Fig), with *B*. *natans* PPOX2 appearing as the earliest eukaryotic branch. The mitochondrial ferrochelatase 2 is derived from the chlorarachniophyte host and is phylogenetically affiliated with heterotrophic stramenopiles (oomycetes), the only representatives of the SAR group in this particular clade (Fig 3).

**Fig 4:**
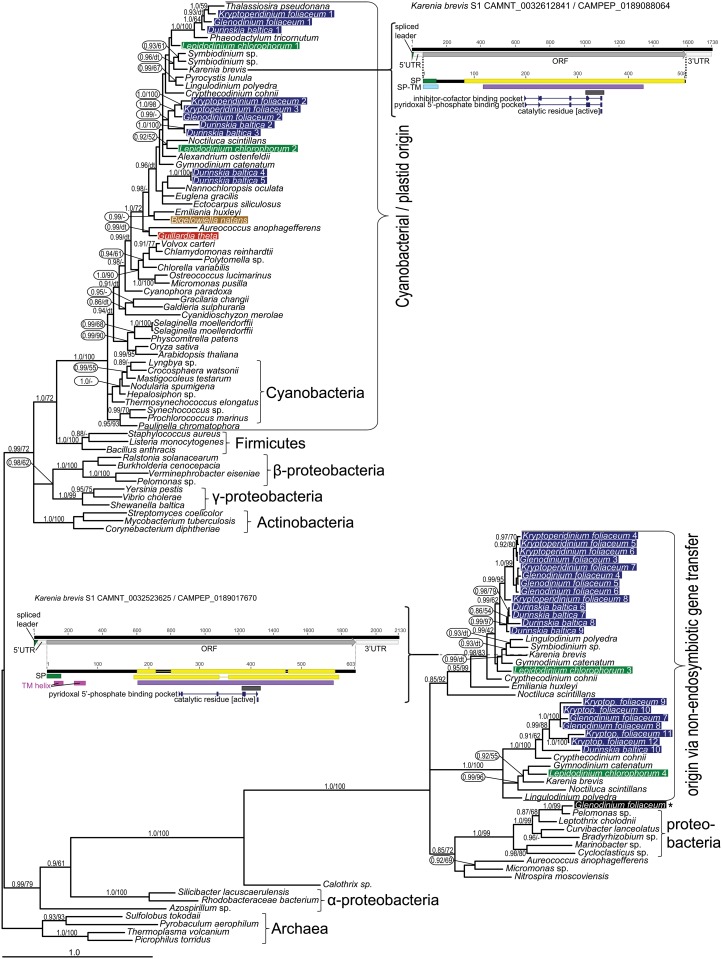
Bayesian phylogenetic tree as inferred from GSA-AT amino acid sequences. Taxa of interest in this study are highlighted by colored bars, blue for dinotoms, green for *Lepidodinium chlorophorum*, ochre for *Bigelowiella natans* and red for *Guillardia theta*. Numbers near branches indicate Bayesian posterior probabilities followed by the bootstrap of respective clades from the maximum likelihood (ML) analysis. Only support values greater than 0.85 (Bayesian) and 50 (ML) are shown. dt—different topology in the ML tree, see S2 Fig; a dash indicates unsupported topology. The tree demonstrates the cyanobacterial origin of canonical GSA-AT, while the non-canonical GSA-AT originates in proteobacteria. Schematics of *Karenia brevis* transcripts and respective proteins are shown for complete representatives of canonical and non-canonical GSA-AT. The presence of a spliced-leader sequence at the 5’ end suggests nuclear encoding and transcription of these genes. An N-terminal presequence of the resulting protein putatively targets both enzymes into the plastid. The canonical and non-canonical enzymes share motifs for pyridoxal 5’-phosphate binding and a catalytical residue. UTR—untranslated region; ORF—open reading frame; TM—transmembrane domain; SP—signal peptide; SP-TM—signal peptide predicted by the SignalP-TM networks; yellow bar—Panther Class III aminotransferase / glutamate-1-semialdehyde 2,1-aminomutase hit; violet bar—Pfam Class III aminotransferase hit; grey bar—PROSITE Class III aminotransferase hit; numbers represent scale in nt or aa.

The autotrophic tetrapyrrole pathway displays mosaic origins in *B*. *natans*, similar to that in other eukaryotic phototrophs. It is mostly composed of cyanobacterial-derived enzymes (glutamate-tRNA reductase BnGTR, glutamate-1-semialdehyde 2,1-aminotransferase BnGSA-AT, BnALAD1, -2, BnPPOX1 and BnFeCH1), but also of enzymes likely originating from the endosymbiont (primary host) nucleus (BnUROD6, BnCPOX1), one enzyme displaying an α-proteobacterial (likely mitochondrial) origin (BnPBGD1), and an additional CPOX (BnCPOX4) of uncertain origin (see Fig 1 for summary). Importantly, many of the aforementioned enzymes show unexpected phylogenetic affiliations: GTR, GSA-AT, ALAD1, CPOX1, -2 and PPOX1 in *B*. *natans* cluster with rhodophytes or algae with red secondary plastids (see Figs 1, 2, 4 and S1 for details), in spite of the chlorophyte origin of the current chlorarachniophyte plastid [62]. Only a single *B*. *natans* enzyme, one of the two ferrochelatases, displays the expected and supported chlorophyte origin (BnFeCH1, Fig 3). Although bootstrap values for the ML analyses are moderately supported, the hypothetical scenarios for red origin of many of these proteins are highly supported by Bayesian inference, which is more robust in analyses of data with high variability across sites. Furthermore, considering the good support for plastid genome relationship with green algae [62], one would expect the clear and well-supported association of *B*. *natans* sequences with either chlorophytes or heterotrophic eukaryotes; this is not observed.

Several enzymes of the autotrophic pathway are present in multiple copies in *B*. *natans*, namely ALAD (Fig 2), UROD and CPOX (S1 Fig). The genome of *B*. *natans* contains two, likely paralagous, genes encoding ALAD, at least one of them affiliated with the red lineage (Fig 2). Three genes coding for autotrophic CPOX have been found in the *B*. *natans* genome, and all three of them appear to be plastid-targeted (S1 Table): one gene (BnCPOX4) is recovered with sequences from other phototrophs with no support; the origin of this clade is unclear. BnCPOX1 appeared within the clade composed of red-derived secondary algae and appeared to be related neither to chlorophytes nor to rhodophytes (S2 Fig). We propose that these genes might originate from the endosymbiont (primary host) nucleus (Figs 1 and S1).

The putative cellular locations corresponding to all enzymes involved in tetrapyrrole biosynthesis in *B*. *natans* are shown in S1 Table, as inferred using SignalP [63] and TargetP [64]. The entire autotrophic tetrapyrrole pathway is likely located in the plastid stroma of the chlorarachniophytes; no enzyme seems to be targeted to the periplastidal space with the possible exception of BnPBGD2, which fulfills some of the criteria described by Curtis et al. [31], namely high number of introns, D/K amino acids at the C terminus, and transit peptide net charge -1. The biological function of an isolated enzyme in this compartment, however, would be unclear and this targeting is unlikely. The plastid-origin BnALAD1 is putatively retargeted to the chlorarachniophyte mitochondrion where it is involved in the mitochondrial-cytosolic pathway. Conversely, paralogs of heterotrophic UROD4 and -5 appear to be retargeted to the plastid compartment (Fig 1 and S1 Table).

*B*. *natans* shares the analogous biparallel architecture of tetrapyrrole biosynthesis with photosynthetic euglenids. Plastid acquisition occurred relatively recently in euglenids, since phototrophic euglenids constitute a monophyletic group [36] and the previous presence of plastids was never confirmed in phagotrophic euglenids or their osmotrophic relatives [37]. In spite of the independent origins of chlorarachniophytes (Rhizaria) and euglenophytes (Excavata) [62], they display a similar pattern of tetrapyrrole synthesis [55]. Both algae possess two nearly complete pathways, one originating from the primary heterotrophic host, the other from the engulfed algal endosymbiont (Fig 1). In both algae the reduction of the redundant pathway has already begun and the mitochondrial-cytosolic pathway is partially reduced. In *Euglena*, two enzymes of plastid origin functionally replaced the original heterotrophic pathway genes for UROS and UROD, either via dual targeting or by sharing the products of the reactions they catalyze between compartments [55]. Similarly, in *Bigelowiella*, the original ALAD and UROS from the heterotrophic pathway were likely replaced via dual localization of the plastid-derived enzymes or through the exchange of pathway intermediates (Fig 1). Furthermore, one of the PBGD enzymes (possibly BnPBGD2) must be dually targeted for the heterotrophic pathway to function. As in other phototrophs, both *B*. *natans* and *E*. *gracilis* contain multiple copies (orthologs) of UROD and CPOX. In summary, the level of metabolic reduction is comparable in *Bigelowiella* and *Euglena*, which suggests that they acquired their green plastids at approximately the same time (assuming similar rates of evolution) or a constraint imposed on cellular metabolism that prevents a loss of redundancy in chlorarachniophytes.

### The ultimate step of the cryptophyte pathway is bifurcated

With the exception of the ferrochelatase predicted to localize in the mitochondrion (see below), only the set of enzymes originating from the algal endosymbiont and putatively targeted to the plastid compartment was found in *Guillardia theta* (Fig 1). We found three copies of UROD (one duplicated cyanobacterial gene and one gene originating from the endosymbiont nucleus) and CPOX (one duplicated gene from the endosymbiont nucleus and one gene of uncertain origin, GtCPOX2; see Figs 1 and S1 and S1 Table); both multiplied sets of enzymes are consequently combinations of orthologs originating from the primary endosymbiotic event (see S1 Fig for details), with one of them duplicated a second time. Gene duplication and functional specialization are also seen in other algae and plants [65]. While the duplication of the cyanobacterial UROD seems to be deeply branching, paralogs of CPOX arose more recently. Most of the enzymes involved originated from a cyanobacterial plastid ancestor related to rhodophytes and the CASH lineage (GtGTR1 and -2, GtGSA-AT, GtALAD, GtUROD1 and -2, GtPPOX; see Figs 2–4 and S1), supplemented by enzymes originating from the endosymbiont nucleus (UROD3, CPOX1 and -3), α-proteobacteria (mitochondria; GtPBGD) and enzymes encoded by algae-affiliated genes of unknown origins (GtUROS, GtCPOX2) (see overview in Fig 1). This is in line with the presence of the rhodophyte-derived secondary plastid in cryptophytes [66,67]. Clustering of GtUROD3 with homologs from green algae, *E*. *gracilis* and sequences from the CASH group (S1 Fig) may be the result of gene duplication followed by lineage-specific gene loss (discussed below).

The mitochondrion-located ferrochelatase GtFeCH2 is eukaryotic in origin, as are its homologs in heterotrophic eukaryotes and the glaucophyte *Cyanophora paradoxa* (Figs 1 and 4), and putatively targeted to the mitochondrion (S1 Table). The retention of mitochondrial ferrochelatase may be the result of slower rates of evolution in the cryptophyte plastid when compared to other red-derived secondary plastids [55–57,68], an evolutionary constraint placed on its role in the biology of the organism, or an independent and more recent plastid acquisition in cryptophytes. The latter view is consistent with the presence of a nucleomorph in cryptophytes and with the growing body of evidence showing that phototrophic cryptophytes emerged through an independent endosymbiosis event [18,69,70].

### Novel type of GSA-AT and a proteobacterial FeCH in dinotoms

We analyzed transcriptomes from the dinotoms (dinoflagellates with tertiary diatom endosymbionts) *Glenodinium foliaceum* CCAP 1116/3, *Kryptoperidinium foliaceum* CCMP 1326 and *Durinskia baltica* (available via the MMETSP initiative, see S3 Table). Two redundant heme pathways are present in dinotoms: one is located in the diatom endosymbiont, while the second remains in the putative remnant of the original peridinin plastid, the eye spot [47]. These separate pathways seem to supply tetrapyrroles to the two independent symbiotic partners. Our inferred trees display similar topologies and evolutionary relationships as those published previously (see Fig 1 for summary; [47]); the endosymbiont pathway enzymes are related to sequences from free-living diatoms, while the host enzymes cluster together with other dinoflagellates. Furthermore, we identified a new biochemically uncharacterized subfamily of putative GSA-AT, which is found in other dinoflagellates as well as in *Aureococcus anophagefferens*, *Emiliania huxleyi* and the chlorophyte *Micromonas*. Since the non-canonical putative GSA-AT clade contains a smaller clade composed of proteobacteria, the possibility of bacterial contamination has to be taken into account. However, spliced-leader sequences in the GSA-AT transcripts from *Karenia brevis* (CAMNT_0032523625 and CAMNT_0032609079, see Fig 4 for details) indicate that the genes are located in the dinoflagellate nucleus. The respective translated sequences contain N-terminal presequences putatively targeting the protein product to the peridinin plastid [71] (Figs 1 and 4). There is also a single sequence from *G*. *foliaceum* closely related to *Pelomonas* sp. in the bacterial cluster; this particular gene might be a bacterial contaminant (Fig 4). The origin of the novel GSA-AT clade remains unclear.

We also analyzed the last enzyme of the pathway, ferrochelatase (FeCH), not included in previously published analyses [47]. Ferrochelatase in particular shows complex origins in eukaryotic phototrophs, which includes non-endosymbiotic gene transfer from proteobacteria to the ancestor of chromerids and apicomplexans (Fig 3). Two clades in the ferrochelatase tree contain dinotoms: the genes of cyanobacterial origin came from the diatom endosymbiont; this clade also contains the heterotrophic dinoflagellate *Oxyrrhis marina*, numerous cyanobacteria, a glaucophyte, the rhizarian *Paulinella chromatophora*, chlorophytes, plants, heterokonts, eustigmatophytes, a haptophyte, *Euglena gracilis* and chromerids. The other clade is sister to apicomplexans, chromerids, and *E*. *gracilis*, and contains rhodophytes, peridinin-pigmented dinoflagellates, and a heterotrophic dinoflagellate (*Cryptheconidium cohni*). Its origin is unclear but might be proteobacterial (Fig 3). The tree topology is consistent with the presence of two ferrochelatases in chromerids [57], phototrophic euglenids [55], and dinotoms. While in apicomplexan parasites the complex origin of ferrochelatase is a result of non-endosymbiotic gene transfer from proteobacteria to Apicomplexa, the ferrochelatases in dinotoms arose from endosymbiotic association with the ancestor of the peridinin plastid and later tertiary endosymbiosis with the diatom endosymbiont.

The heme pathway is redundant in dinotoms; however, there are putatively necessary genes missing from their transcriptomes (Fig 1). The most striking absence is that of dinoflagellate-like UROS. We are unable to unambiguously discriminate between endosymbiont and host nuclear-encoded enzymes based solely on their sequences as spliced leaders are often missing; still, it appears that the diatom-like enzymes are exclusively used by the diatom endosymbiont, mainly due to retained characteristics required for diatom-like protein transport (the ASAFAP motif [47,72]) as well as difficulties in the hypothetical transport of proteins from the host cytoplasm over 6 membranes (endomembrane system of the host + putative plasma membrane of endosymbiont + four membranes of the diatom plastid). Conversely, any transport of UROS from inside the diatom endosymbiont compartment to the eyespot (remnant of the original dinoflagellate peridinin plastid) is hard to imagine. The absence of the original dinoflagellate UROS could be explained by transport of pre-uroporphyrinogen (hydroxymethylbilane), however, pre-uroporphyrinogen is highly unstable [73]. Furthermore, all the antecedent enzymes in the eyespot pathway (GTR, GSA-AT, ALAD, PBGD) would become redundant and therefore should have been lost from the genome. Insufficient sequencing and high divergence of UROS may explain the total absence of transcripts of the dinoflagellate-like UROS. On the other hand, the absence of *Glenodinium* orthologs of KfUROD1, KfUROD4 and the KfCPOX3+4+5 cluster and the *Kryptoperidinium* ortholog of the GfCPOX3+4 cluster (S1 Fig) may also be a result of gene loss, as other functional copies remained. In several cases, sequencing and assembly errors may interfere with determining the exact number of closely related paralogs, GSA-AT being an example of high gene copy number in dinoflagellates (Fig 4).

### The rhodophyte pathway is conserved in algae with green plastids

Using our transcriptomic data (S2 Table), we mapped the tetrapyrrole pathway in the green dinoflagellate *Lepidodinium chlorophorum* [24,74]. We found most of the genes of the plastid tetrapyrrole pathway and no traces of the mitochondrial-cytosolic pathway (see Fig 1 for summaries), consistent with the autotrophic history of the species. Some enzymes (LcGTR, LcPPOX2 and LcFeCH) cluster with the red lineage (S1 Fig). Other sequences branch with green algae and plants but always also branch together with other dinoflagellate sequences (LcALAD, LcGSA-AT1, -2, LcPPOX1). For example, sequences of canonical GSA-AT from *L*. *chlorophorum* form a cluster with sequences from the red lineage (Fig 4); however streptophytes, chlorophytes and rhodophytes are unresolved and placed in the ancestral position (see Figs 4 and S1 for details).

Interpreting most phylogenetic trees is very complicated, mainly due to the existence of multiple genes originating in the host nucleus, the endosymbiont nucleus, cyanobacteria (plastid), and proteobacteria. Together these factors make the evolution of heme pathway enzymes difficult to follow, particularly in eukaryotic phototrophs. However, the phylogenetic placement of *L*. *chlorophorum* GSA-AT and other enzymes together with their orthologs from dinoflagellates with rhodophyte-derived plastids suggests a red origin. In LcALAD and LcPPOX, with affinities to green algae, we presume the topology could be the result of the duplication of cyanobacteria-derived genes and subsequent lineage-specific gene loss (Fig 2), similar to GtUROD3. In other words, the hypothetical ancestor of the plastid contained two paralogs, for clarity here denoted as red and green, and one these paralogs was later lost in each lineage (the green one in rhodophytes and stramenopiles, the red one in the green lineage and dinoflagellates), masking the true origin of the gene. A clear example is seen in PPOX: the gene passed through a duplication event (S1 Fig) and *L*. *chlorophorum* genes are present in both cyanobacterial PPOX clades, with either rhodophytes or chlorophytes at the root. Again, they group together with other algae possessing rhodophyte-derived plastids. This suggests the green paralog was inherited vertically, not via endosymbiotic gene transfer from the green endosymbiont. It is noteworthy that the paralogs of ALAD and PPOX retained in rhodophytes also remained in stramenopiles, haptophytes and dinotoms, while the genes found in green algae and plants are present only in dinoflagellates. Consequently, most of the “red-related” genes in dinotoms apparently originate from, and reside in, the diatom endosymbiont (Figs 1 and 2, S1) and the ancestral dinoflagellate genes appear “green-related”. An alternative hypothesis would imply horizontal (eukaryote-to-eukaryote) gene transfer of ALAD and PPOX from green algal prey or from a putative green plastid to the ancestor of dinoflagellates, to the exclusion of chromerids and apicomplexans that possess a red-related gene. This putative green plastid would appear cryptic from today`s perspective, as it must have been later functionally replaced and partially genetically masked by the current red-derived peridinin plastid.

Therefore, it appears that most of the enzymes considered here originate from a rhodophyte source, in spite of the dinoflagellate’s chlorophyte-derived plastid. The chlorophyte-derived plastid is thought to have replaced the original peridinin pigmented dinoflagellate plastid through serial secondary endosymbiosis in this species [23]. Regardless, we observed that the original rhodophyte-derived pathway introduced with the peridinin plastid is highly conserved in this dinoflagellate, in agreement with the “shopping bag” or plastid promiscuity hypothesis, resulting in a mosaic evolution of the plastid proteome [75]. This functional conservation of tetrapyrrole biosynthesis genes might result from a predisposition of red genes to be targeted to the new plastid (as they were already successfully targeted to the old one)–an advantage the newcomer green genes presumably lacked.

**Fig 5:**
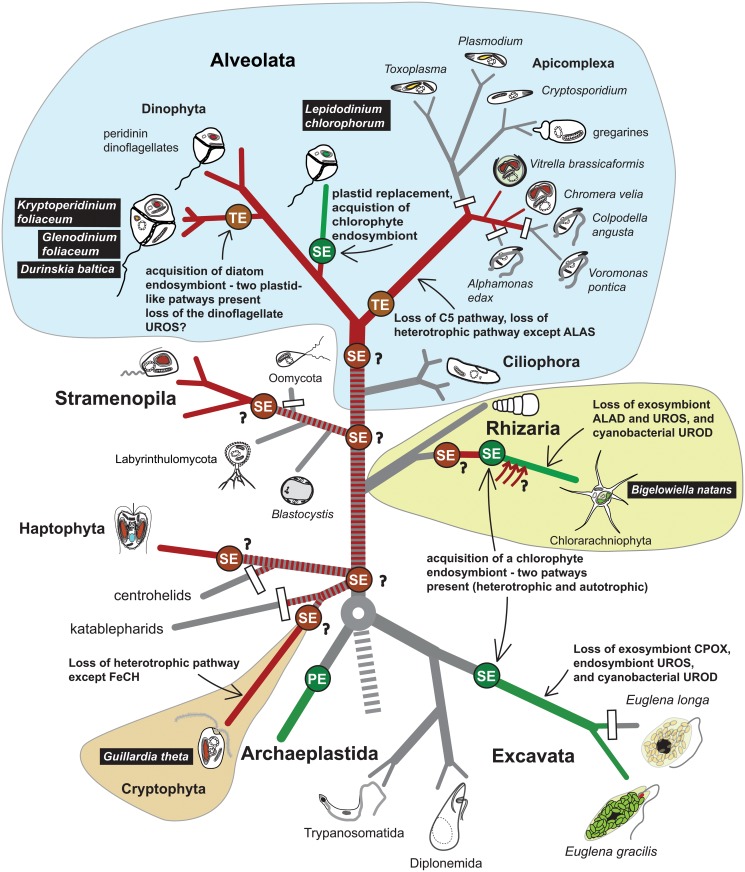
A simplified scheme of the evolution of the tetrapyrrole biosynthesis pathway with an emphasis on the models from this study (black bars). Primary endosymbiosis (PE) gave rise to the Archaeplastida comprising red algae, green algae and glaucophytes. Following the divergence of main eukaryotic lineages, secondary (SE) or tertiary endosymbiosis (TE) events equipped the ancestors of CASH taxa (cryptophytes, alveolates, stramenopiles and haptophytes) with photosynthetic capabilities. Contradictory evidence has been debated over the last years as for the history of CASH plastid acquisitions (e.g. [5,8,34]). A plastid-early scenario (the chromalveolate hypothesis) posits that all CASH taxa are monophyletic and the CASH plastid was vertically transferred (dashed red line) and lost in extant non-photosynthetic descendants (such as ciliates and most rhizarians). Plastid-late scenarios require multiple lateral acquisitions of the CASH plastid (question marks) but better reflect some current phylogenomic analyses of the plastid recipients (e.g. [18,69]). Loss of photosynthesis/plastids have been documented in many sister lineages, such as oomycetes or apicomplexans, however these are in line with plastid-late scenarios as well. A cryptic SE with a CASH alga or numerous HGT (red arrows) events are inferred before the divergence of extant chlorarachniophytes (this work, [78]), which was masked by the acquisition of the current green algal endosymbiont. A similar situation in *L*. *chlorophorum* led to the peridinin plastid replacement with a green plastid, however the majority of red-related heme pathway enzymes remained functionally conserved in the successor plastid. The loss of the heterotrophic pathway possibly occurred several times independently in the stramenopile and dinoflagellate lineages, as *Perkinsus marinus*, sister to dinoflagellates, still contains a functional mitochondrial-cytosolic pathway [8].

The predominantly rhodophyte origins of the heme pathway in *L*. *chlorophorum* likely represent a set from the previously acquired peridinin plastid, but the “purchased” old genes were put into a newer, better shopping bag. The presence of red-derived heme pathway enzymes in *B*. *natans* plastids could be a result of non-endosymbiotic HGT from (red) algal prey according to the “you are what you eat” hypothesis [76]. Indeed, a rich fraction of red-related genes in *B*. *natans*, including photosynthesis-related genes, has already been reported [77,78]. Additional photosynthesis-related proteins were recruited from bacteria, indicating that extensive HGT does take place in chlorarachniophytes [77]. One explanation for the ease of HGT in this case comes from the origin of heme pathway genes; *B*. *natans* sequences often cluster with the CASH taxa. If these genes were horizontally transferred from one of the CASH lineages, they already coded for some plastid targeting signal as heme synthesis is presumably plastid-located in these lineages. *Bigelowiella natans* does not use the same protein import complex (SELMA) as algae from the CASH taxa [79], but plastid proteins in these groups have some structural similarities (e.g. the presence of a bipartite N terminal extension comprising a signal peptide plus a transit peptide) suggesting similarities also in the protein transport mechanisms. On the other hand, the abovementioned HGT events from the red lineage must have taken place after the green plastid acquisition but before the divergence of two basal chlorarachniophyte lineages comprising *Lotharella amoeboformis* and *B*. *natans* [78]. Altogether, it seems less probable that massive gene replacement via non-endosymbiotic HGT would occur in enzymes forming an essential and compartmentalized metabolic pathway. Taking into account similarities with *L*. *chlorophorum*, we can speculate that the green dinoflagellate “heritage” scenario also applies to *B*. *natans*. The rhodophyte origin of the tetrapyrrole pathway in *B*. *natans* may therefore reflect the previous presence of a hypothetical red-derived plastid in the ancestor of chlorarachniophytes (Fig 5). Rhizarians exhibit predatory heterotrophic or parasitic lifestyles, and a cryptic plastid could be held initially as a kleptoplast [80]. Similarly in dinoflagellates, the origins of “green” ALAD and PPOX may trace back to a cryptic endosymbiosis or gene transfer from the green lineage in the common ancestor of extant dinoflagellates including *Oxyrrhis* (Fig 2), mirroring the gene flow from the red lineage observed in *B*. *natans* [78]. However, the number of genes significantly related to the green lineage is extremely limited in studied dinoflagellates and *Chromera velia* [33,81,82] and does not necessarily imply the cryptic introduction of a green plastid. Indeed, the observed topologies might be artifacts showing false phylogenetic affinities. However, considering balanced sampling of each higher taxon (CASH taxa, green algae plus plants, red algae), this is less likely to happen in all cases.

In order to determine how endosymbiotic events occurred, a robust reconstruction of the gene repertoire of photosynthetic algae is needed. Not all genes diverged at the same time and gene multiplication and lineage-specific losses may hinder phylogenetic signal resolution. Indeed, genes with conserved or ancient evolutionary histories display different topologies than those acquired more recently via HGT [34] or those possessing less conserved functions [83]. Curtis et al. [31] reported a high number of algal-related genes in *G*. *theta* that acquired new functions and putatively also cellular localizations through endosymbiotic gene transfer to the host nucleus, regardless of their evolutionary origin. This is in conflict with the conserved origins implicated in this study; we suggest that enzymes of essential plastid pathways, such as tetrapyrrole biosynthesis, resist functional replacement due to conserved localization to a specialized compartment. The protein transport mechanism (SELMA), present in all investigated CASH taxa, is strong molecular evidence for the monophyletic origin of the CASH plastid [5,6,14]. During the course of evolution, proteins transported into the plastid via this mechanism have acquired an N-terminal transport signal. This potentially enables their lateral movement to new eukaryote hosts and allows them to maintain their original functions inside the organelle, provided the same transport mechanism is employed in the new host. This is consistent with theories of lateral CASH plastid transfer into unrelated branches of the eukaryote tree, giving rise to the CASH taxa [34]. Conversely in cases of serial plastid replacement, a potential new plastid would encounter a pre-existing, functional set of proteins ensuring the function of the original organelle. The new plastid might also be inhabited by the original plastid’s protein compendium including the protein transport machinery, rather than continuing to use its own proteome, which would be incompatible with SELMA. With this in mind, we presume that proteins having the ability to be transported to the CASH plastid could also be transported into a successor plastid, enabling the detection of serial endosymbiotic histories with higher confidence than cytoplasmic proteins.

## Conclusions

The tetrapyrrole biosynthesis pathway in phototrophic eukaryotes is an evolutionary mosaic originating in proteobacteria, cyanobacteria and eukaryotes. It represents a shopping bag of enzymes collected during the history of plastid endosymbiosis retained, due to its essential role in metabolism, even after photosynthetic capabilities have been lost. Here we confirm that the tertiary plastids of dinotoms represent largely independent compartments with tetrapyrrole biosynthesis occurring parallel to biosynthesis in the peridinin plastid. The enzymes putatively localized to the former plastid branch sister to dinoflagellate enzymes, while the tertiary plastid contains enzymes branching sister to those of diatoms, mirroring the origin of the respective organelles. In *G*. *theta*, the pathway is located almost entirely in the plastid, with the exception of a eukaryotic ferrochelatase apparently localized to the mitochondrion, indicating either a slow evolutionary rate or an evolutionary constraint. Furthermore, we observed that the majority of the pathway is evolutionarily conserved and related to the red lineage even in organisms that currently possess a plastid of green algal provenance, i.e. the dinoflagellate *Lepidodinium chlorophorum* and the chlorarachniophyte *B*. *natans*. Hence, if the protein targeting machinery is compatible with the new plastid compartment, the tetrapyrrole synthesis pathway can be relocated “as is”, which is illustrated in the case of *L*. *chlorophorum*. Intriguingly, such a scenario may imply the existence of a cryptic red-derived plastid earlier in the history of chlorarachniophytes. While the evolution of eukaryotes is becoming clearer with increasing data from deeper lineages, the history of plastid acquisitions resists revealing an unequivocal scenario due to massive gene transfer and phylogenetic bias. We suggest that a targeted approach directed at conserved processes could result in new, relevant hypotheses even in the genomic era.

## Material and Methods

The complete genomic sequences of the cryptophyte alga *Guillardia theta* (http://genome.jgi.doe.gov/Guith1/Guith1.home.html) and the chlorarachniophyte *Bigelowiella natans* (http://genome.jgi.doe.gov/Bigna1/Bigna1.home.html) were searched using BLAST [84] for genes encoding enzymes involved in the synthesis of tetrapyrroles (ALAS, GTR, GSA-AT, ALAD, PBGD, UROS, UROD, CPOX, PPOX, and FeCH). Homologous sequences were those used in Kořený *et al*. [57]; newly added sequences are listed in S3 Table. All alignments were made using MUSCLE [85] and ambiguous regions were removed in SeaView [86]. Phylogenetic trees were constructed using Maximum Likelihood (RAxML v8.2.4; [87]), Bayesian inference (PHYLOBAYES v3.3b; [88]) and a method designed to deal with amino acid saturation (AsaturA v18.10.2002; [89]). ML trees were computed using the LG model with gamma distribution in 4 categories and 1000 replicates. Bayesian inferences were calculated with the following parameters: 2 chains, 15,000 generations under the C20 model with Poisson exchange rate, sampling every 100 generations, and a maximum divergence of 0.1.

Sequences from *G*. *theta* and *B*. *natans* were inspected for the presence of N-terminal leader sequences by SignalP 3.0 [63] and TargetP [64], predicting localization to either the mitochondrion (mitochondrial transit peptide) or the plastid (bipartite leader composed of ER signal peptide followed by a transit peptide). GSA-AT of *Karenia brevis* (Fig 4) were automatically annotated using the InterProScan feature of Geneious 8.1 [90].

The transcriptome library of *Lepidodinium chlorophorum* (Roscoff collection no. RCC1488) was generated using the NEBNext Ultra Directional RNA Library kit (New England Biolabs, Ipswitch, MA, USA) according to the manufacturer`s instructions. Quality assessment and sequencing were performed in a specialized facility, using the Illumina MiSeq (2×250 bp) platform. The generated reads were quality-trimmed using the FASTQ Toolkit (v1.0) of the Illumina BaseSpace platform and then assembled using Trinity v2.1.1 [91] and SOAPdenovo2 v2.0 r240 [92] and clustered using CAP3 [93]. Gene assembly completion was assessed with BUSCO software using the complete eukaryotic gene profile [94] on protein models generated by the TransDecoder script of the Trinity package. Some characteristics of the obtained transcriptome are listed in S2 Table. Novel sequences of interest were deposited in GenBank under the accession no. KX344033-47.

## Supporting Information

S1 Fig Bayesian phylogenetic trees as inferred from the amino acid sequences. A) ALAS, B) GTR, C) PBGD, D) UROS, E) UROD, F) CPOX and G) PPOX. Taxa of interest of this study are highlighted by colored bars: blue for dinotoms, green for *Lepidodinium chlorophorum*, ochre for *Bigelowiella natans* and red for *Guillardia theta*. The tree demonstrates the mitochondrial origin of ALAS. Numbers near branches indicate Bayesian posterior probabilities followed by the bootstrap of respective clades from the likelihood analysis. Only support values greater than 0.85 (Bayesian) and 50 (likelihood) are shown. dt—different topology in the likelihood tree, see S2 Fig; a dash indicates unsupported topology. Asterisks mark possible contaminations. LcPPOXa, -b, -c; LcUROSa, -b = non-overlapping protein models, putatively fragments of LcPPOX1 and LcUROS.
(PDF)

S2 Fig Maximum likelihood trees as inferred from amino acid sequences. Numbers near branches indicate bootstrap values; only support values greater than 50 are shown. A, ALAS—delta-aminolevulinic acid synthase; B, GTR—glutamate-tRNA reductase; C, GSA—glutamate-1-semialdehyde aminotransferase; D, ALAD—aminolevulinic acid dehydratase; E, PBGD—porphobilinogen deaminase; F, UROS—uroporphyrinogen synthase; G, UROD—uroporphyrinogen decarboxylase; H, CPOX—coproporphyrinogen oxidase; I, PPOX—protoporphyrinogen oxidase; J, FeCH—ferrochelatase. LcPPOXa, -b, -c; LcUROSa, -b = non-overlapping protein models, putatively fragments of LcPPOX1 and LcUROS.
(PDF)

S1 Table Targeting presequences in *Bigelowiella natans* and *Guillardia theta*. Targeting probabilities were determined using SignalP and TargetP as described in Materials and Methods. Respective targeting peptide sequences are listed. The presence of a signal peptide followed by a chloroplast targeting peptide (cTP) implies localization to the plastid; mitochondrial enzymes encode mitochondrial targeting peptide presequences, while cytoplasmic enzymes lack presequences. If available, models with longer N-termini (e.g. Pasa, Fgenesh) were included in pre-sequence analysis and are listed in the table.
(PDF)

S2 Table Characteristics of the obtained transcriptome libraries of *Lepidodinium chlorophorum*. Number of reads and bases of two libraries are listed after quality trimming (two reads per library, see Material and Methods). The resulting number of contigs and coding sequences were analyzed using the BUSCO pipeline with a set of 429 BUSCO groups of orthologs. Ortholog counts: C = complete; D = duplicated; F = fragments; M = missing.
(PDF)

S3 Table List of sequences added to the original datasets of Kořený *et al*. [26, 28]. Gene copy designation for species of interest in this study is shown in brackets according to their designation in respective trees (an asterisk marks putative contaminant sequences). Databases: CGP = Cyanophora Genome Project; CryptoDB = Cryptosporidium Genomic Resource; gb = GenBank; Gruber ea. = Gruber *et al*. 2015 Plant J, 10.1111/tpj.12734; jgi = DOE Joint Genome Institute; MMETSP = Marine Microbial Eukaryote Transcriptome Sequencing Project; Nori = NoriBLAST, Porphyra Genome Project; psb = bioinformatics.psb.ugent.be; VH = Courtesy of Vladimír Hampl, unpublished; Cmb = combined samples.
(PDF)

## Abbreviations

ALA: δ-aminolevulinic acid
ALAD: ALA dehydratase
ALAS: ALA synthase
CASH: group of complex algae with red-derived plastid of putatively common origin, comprising cryptophytes, alveolates, stramenopiles and haptophytes
CPOX: coproporphyrinogen oxidase
FeCH: ferrochelatase
GSA-AT: glutamate-1-semialdehyde 2,1-aminotransferase
GTR: glutamate-tRNA reductase
HGT: horizontal gene transfer
PBGD: porphobilinogen deaminase
PPOX: protoporphyrinogen oxidase
UROD: uroporphyrinogen decarboxylase
UROS: uroporphyrinogen-III synthase
