# Supplementary figures and images for: Evolution of the Tetrapyrrole Biosynthetic Pathway in Secondary Algae: Conservation, Redundancy and Replacement
Source: PLoS One. 2016 Nov 18;11(11):e0166338. doi: 10.1371/journal.pone.0166338 (PMC5115734; doi:10.1371/journal.pone.0166338)

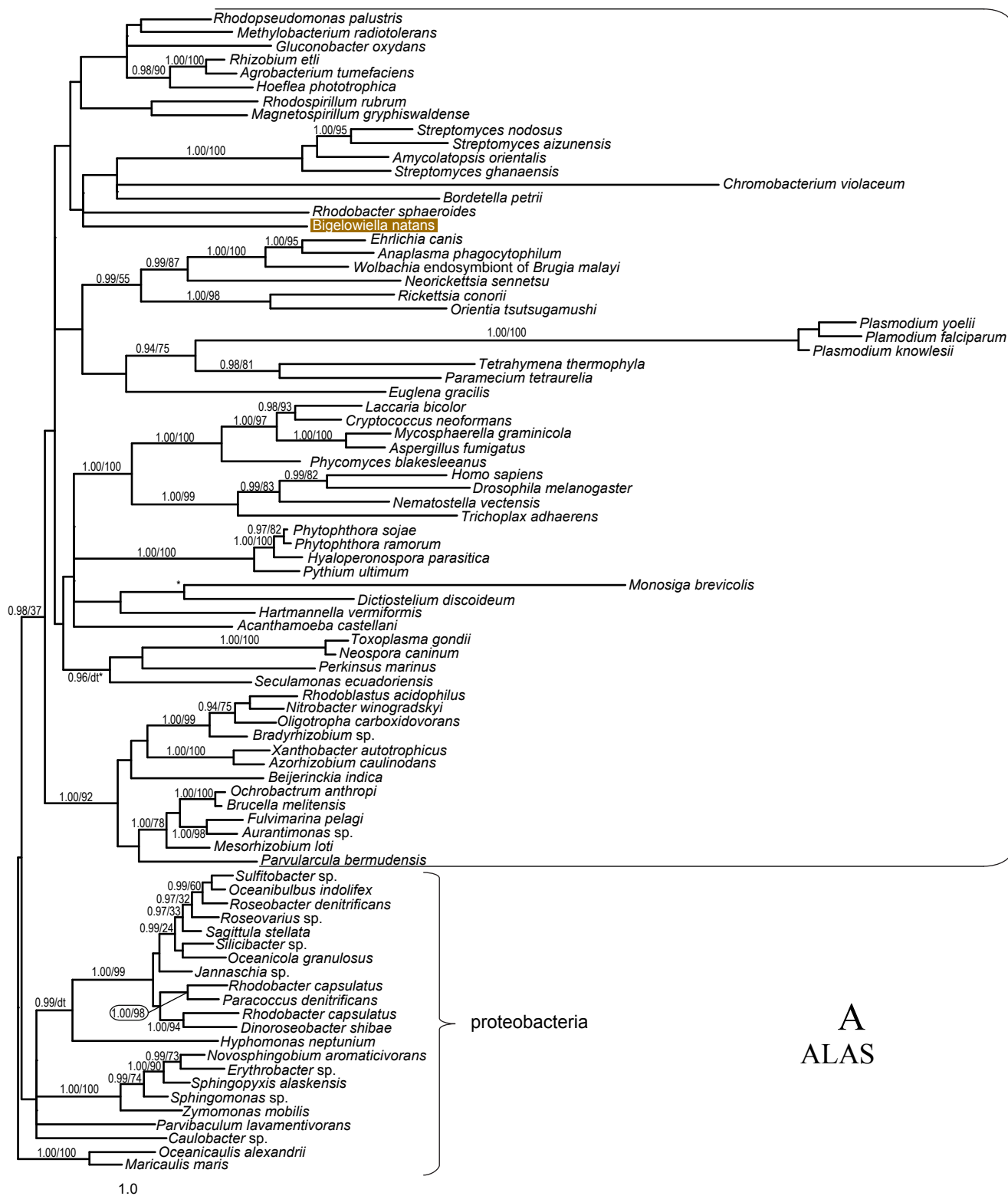

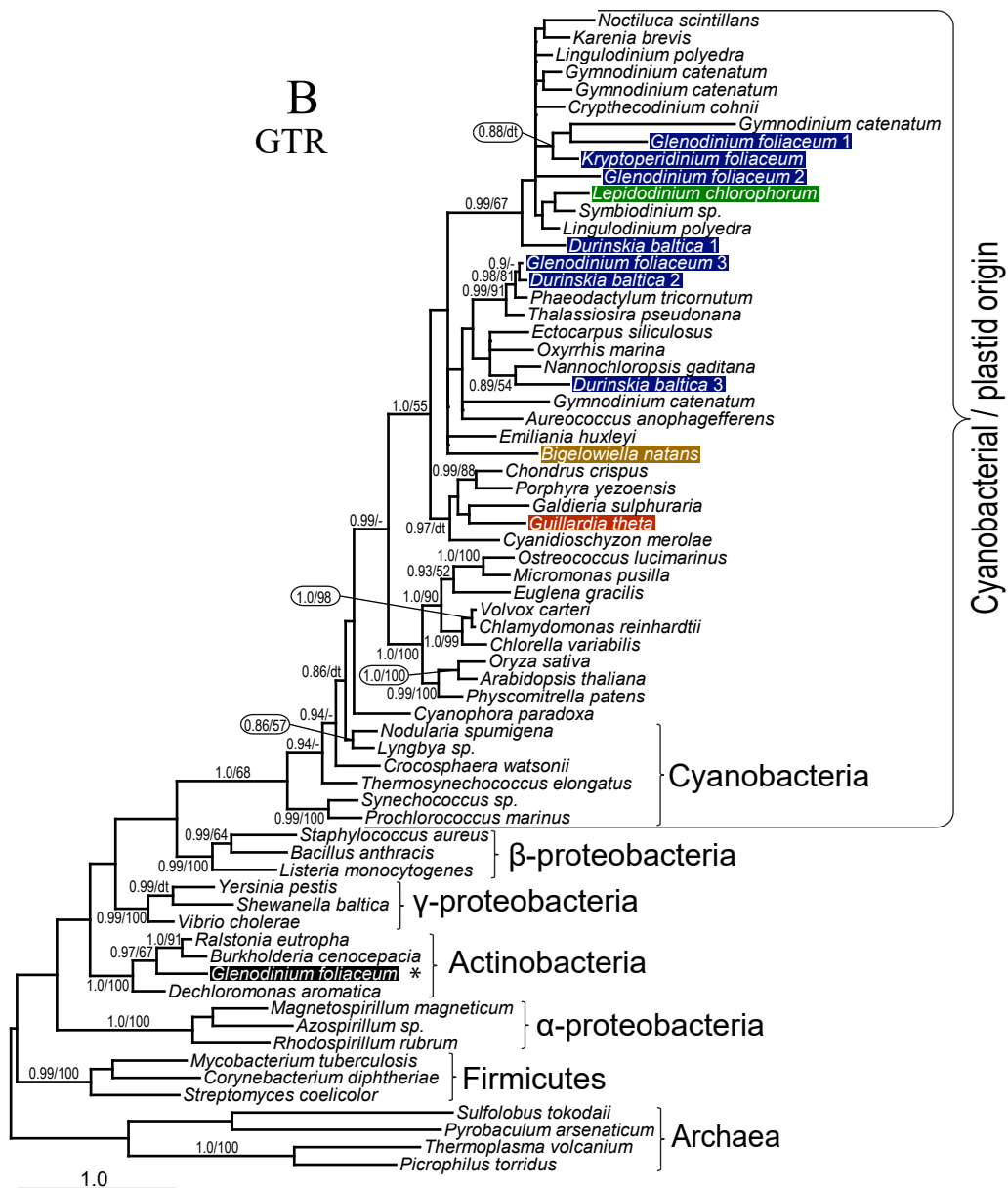

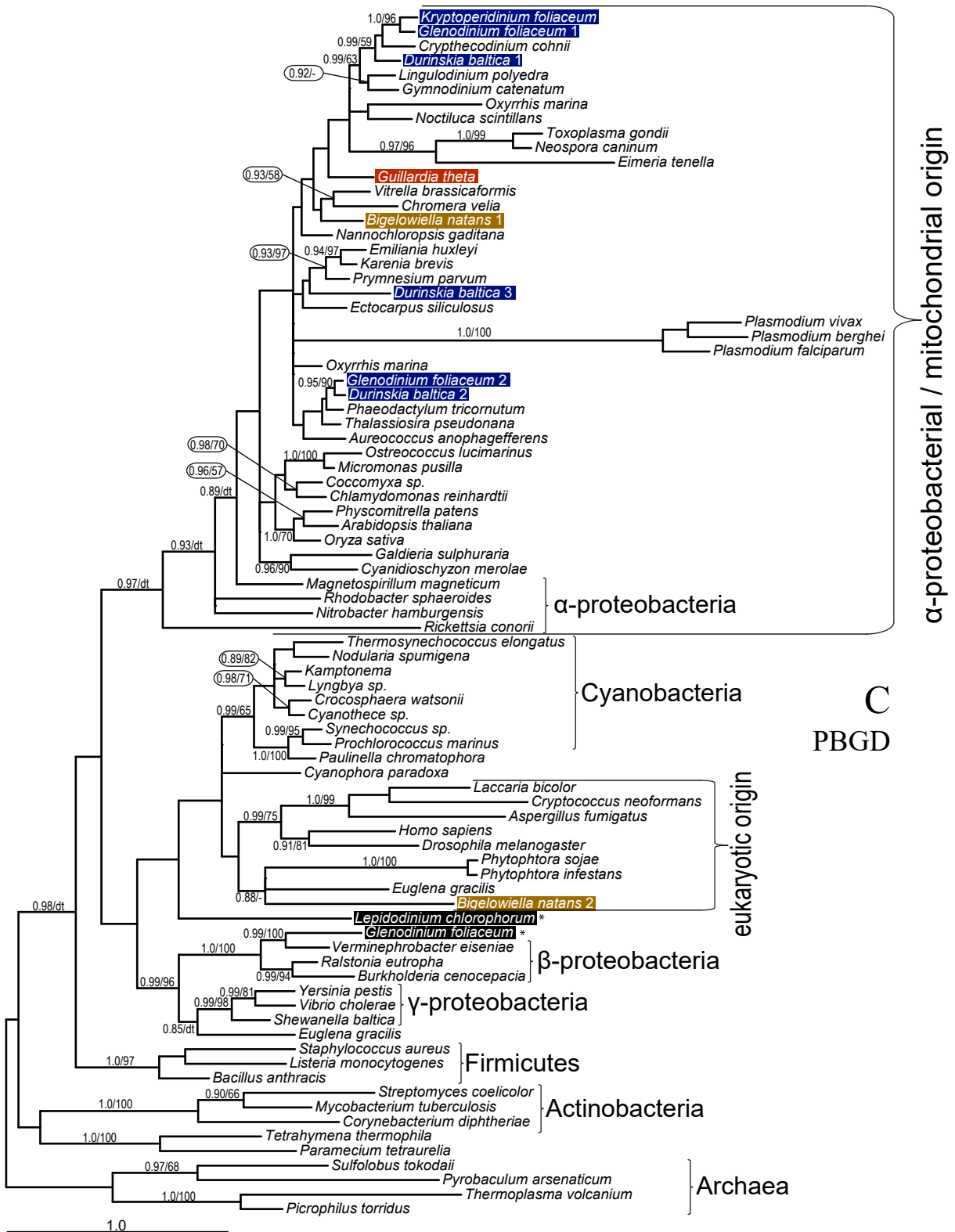

D  
UROS

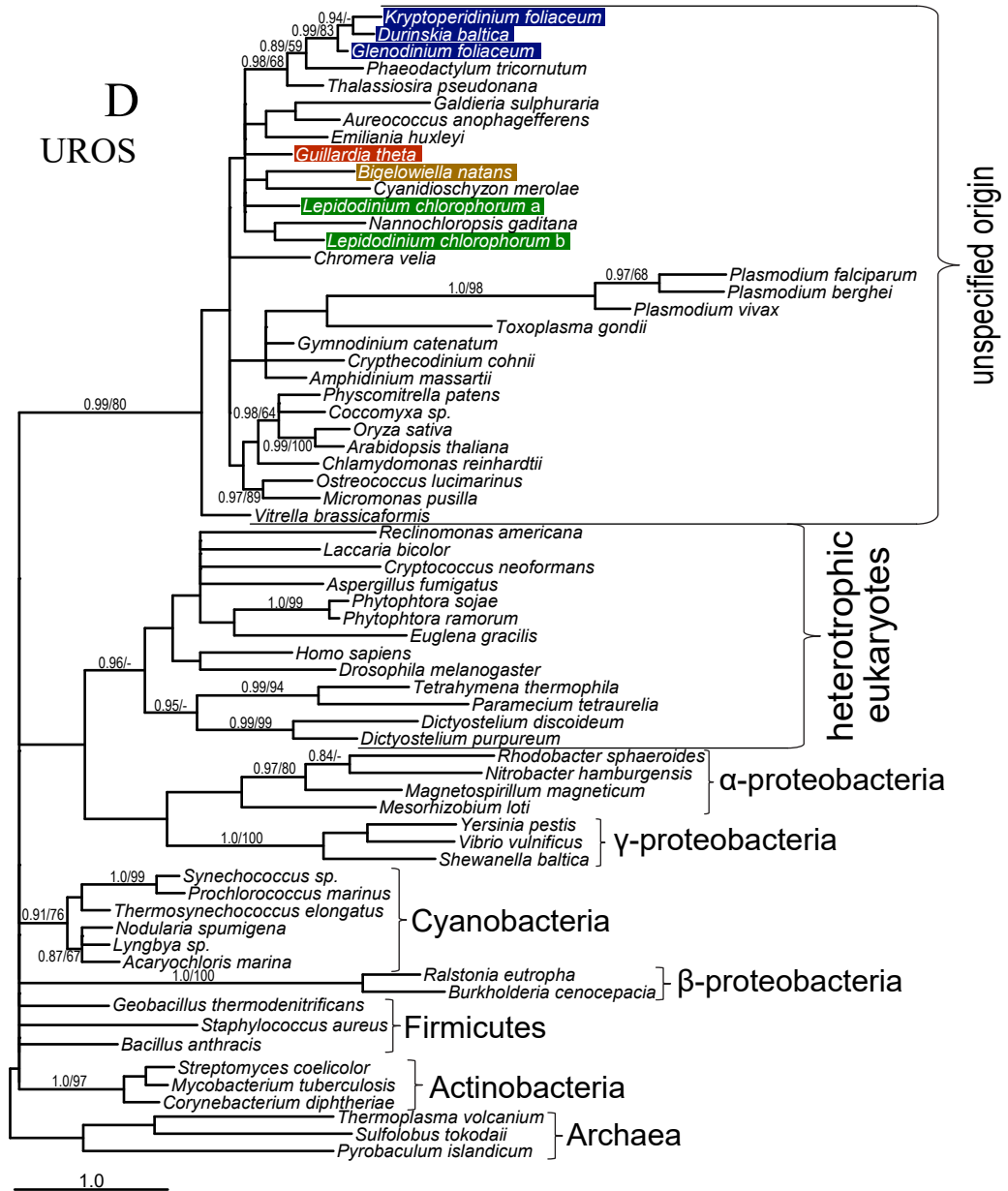

E  
UROD

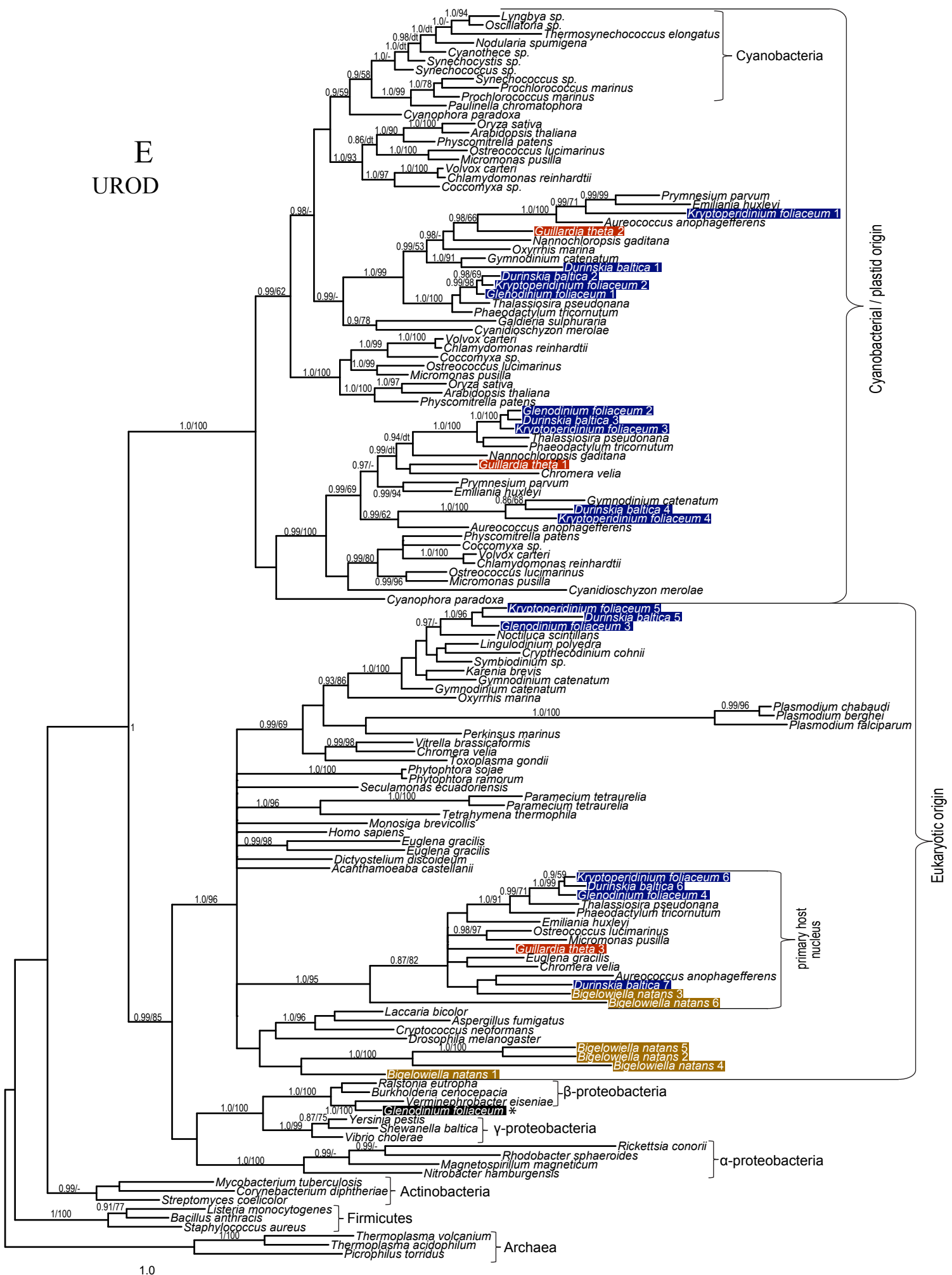

F  
CPOX

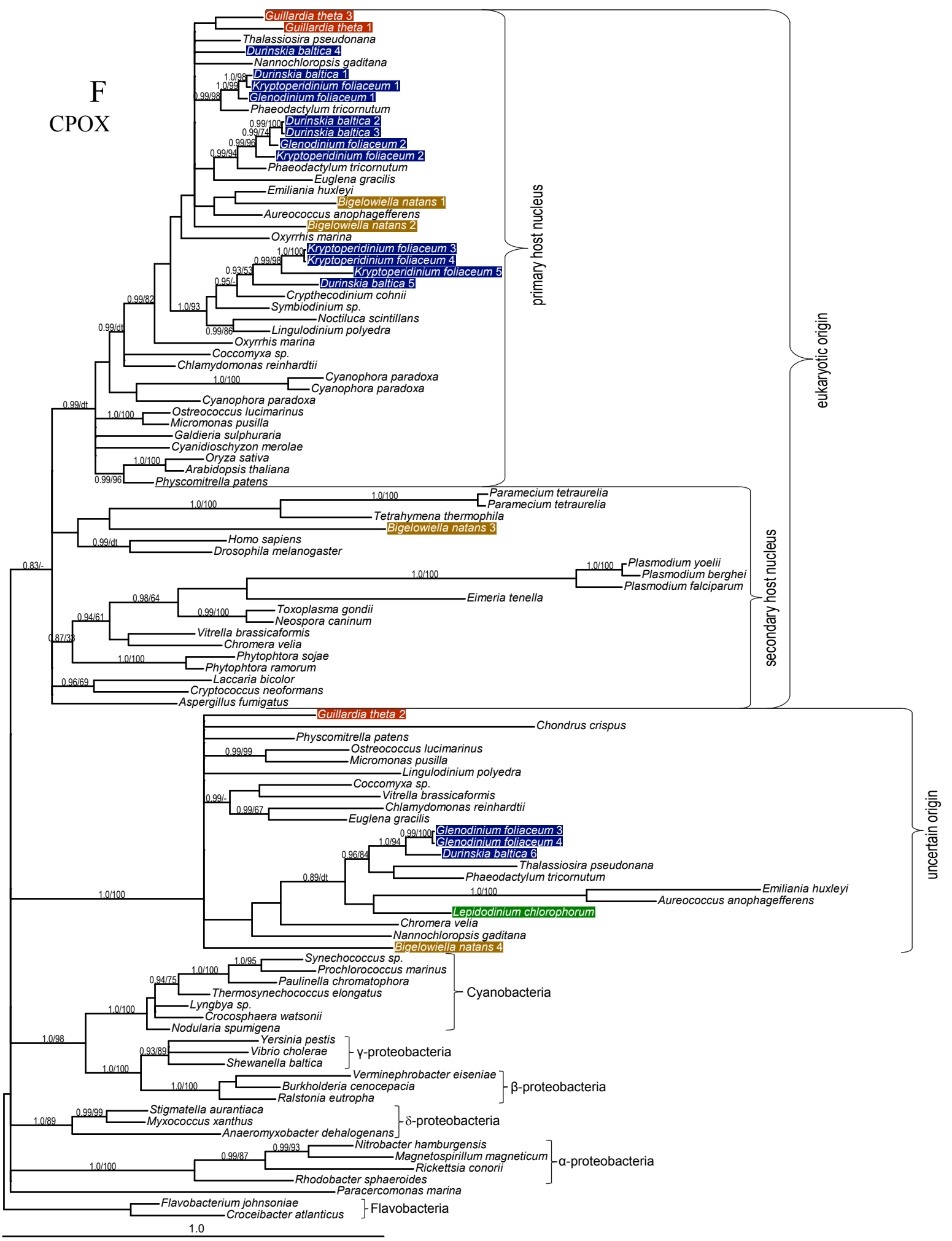

G  
PPOX

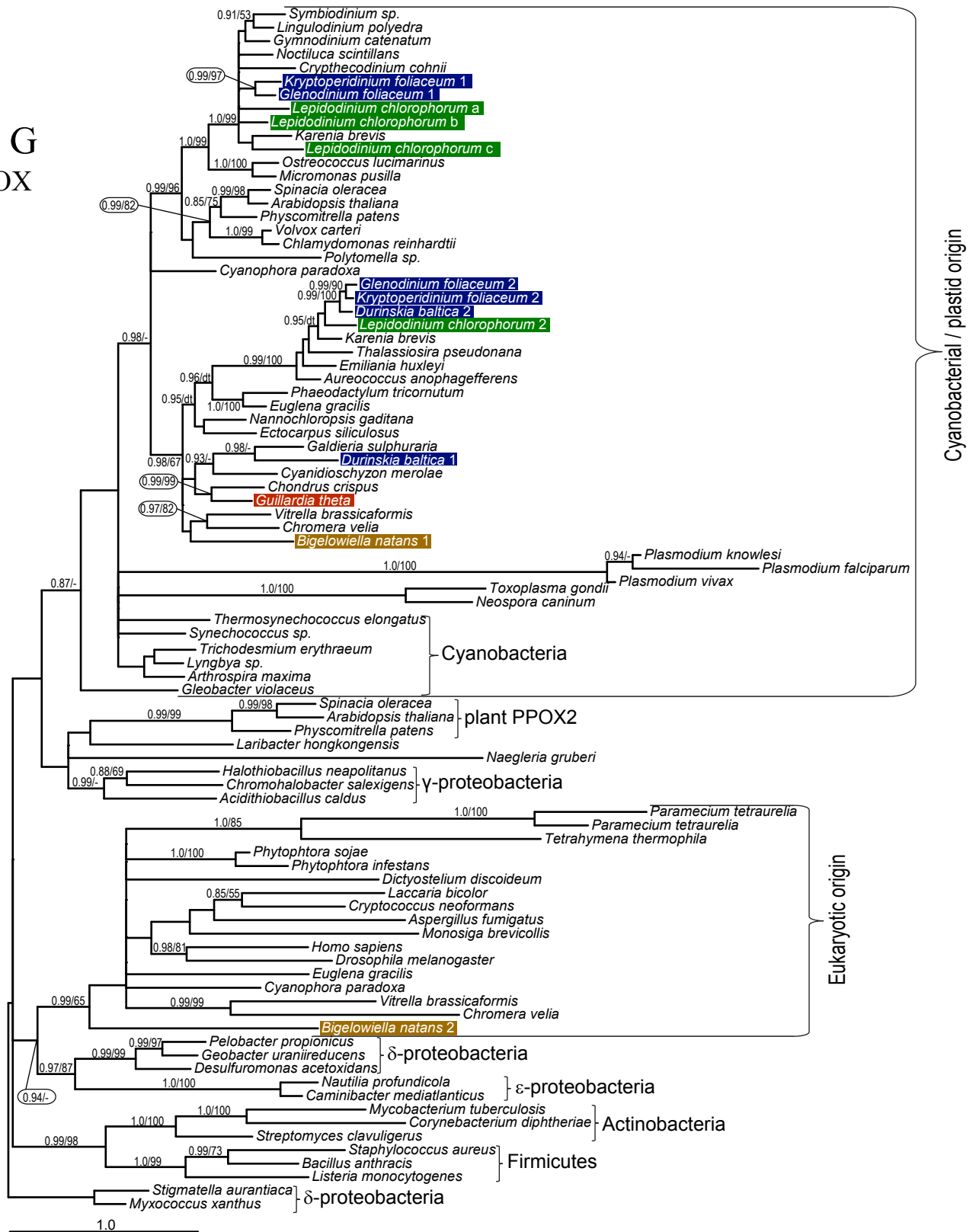

Supplement: S1 Fig — A) ALAS, B) GTR, C) PBGD, D) UROS, E) UROD, F) CPOX and G) PPOX. Taxa of interest of this study are highlighted by colored bars: blue for dinotoms, green for Lepidodinium chlorophorum, ochre for Bigelowiella natans and red for Guillardia theta. The tree demonstrates the mitochondrial origin of ALAS. Numbers near branches indicate Bayesian posterior probabilities followed by the bootstrap of respective clades from the likelihood analysis. Only support values greater than 0.85 (Bayesian) and 50 (likelihood) are shown. dt—different topology in the likelihood tree, see S2 Fig; a dash indicates unsupported topology. Asterisks mark possible contaminations. LcPPOXa, -b, -c; LcUROSa, -b = non-overlapping protein models, putatively fragments of LcPPOX1 and LcUROS. (PDF) [file pone.0166338.s001.pdf]

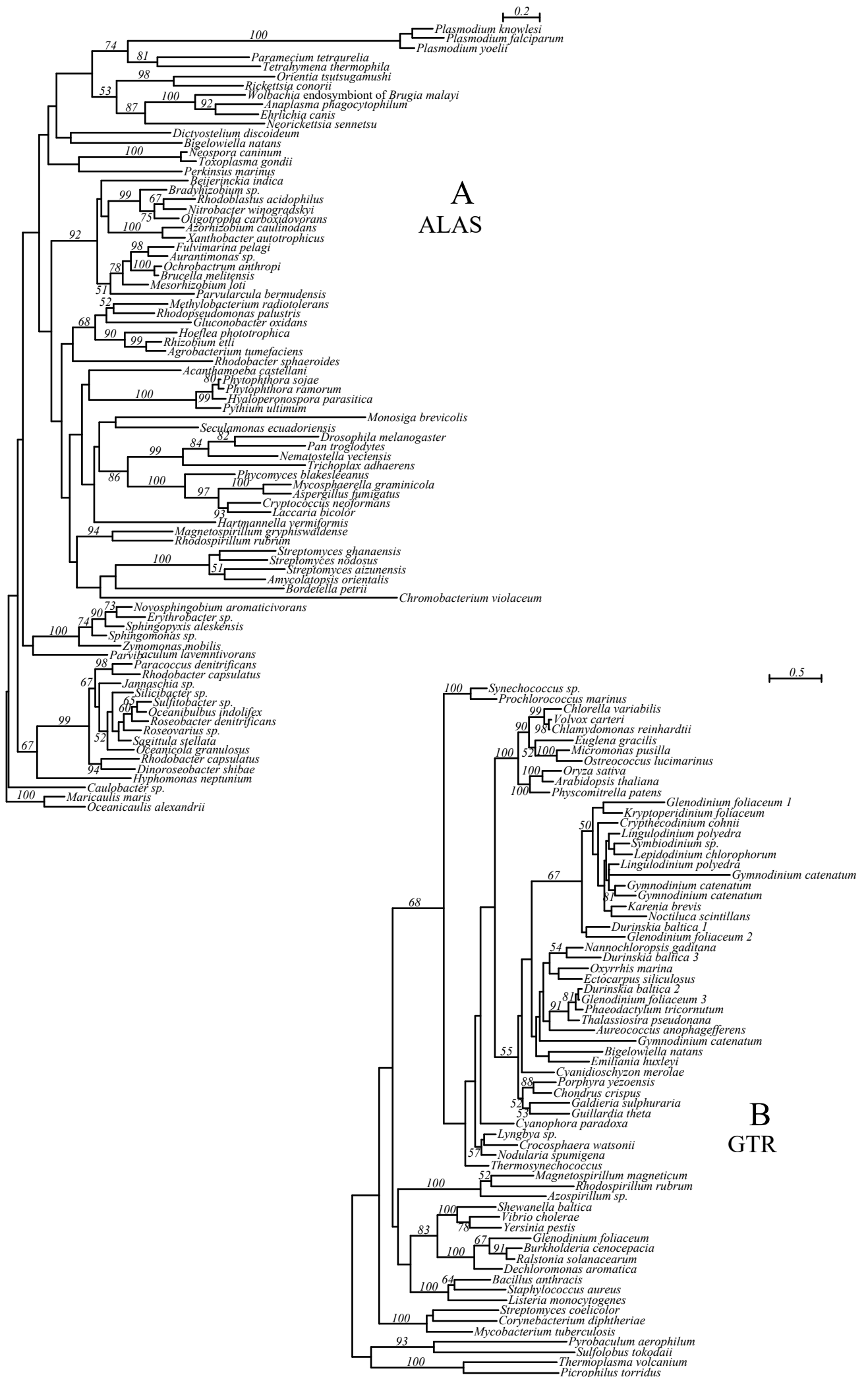

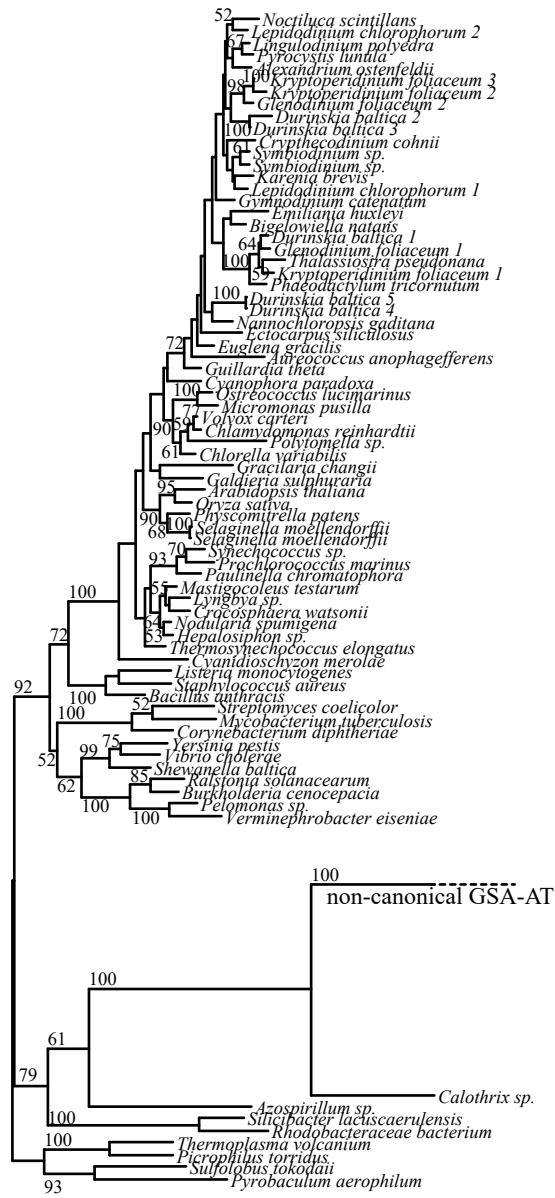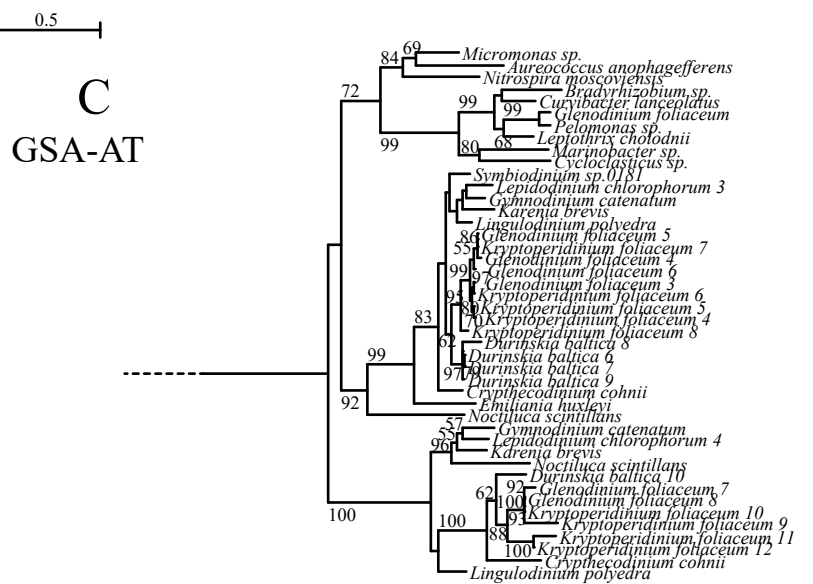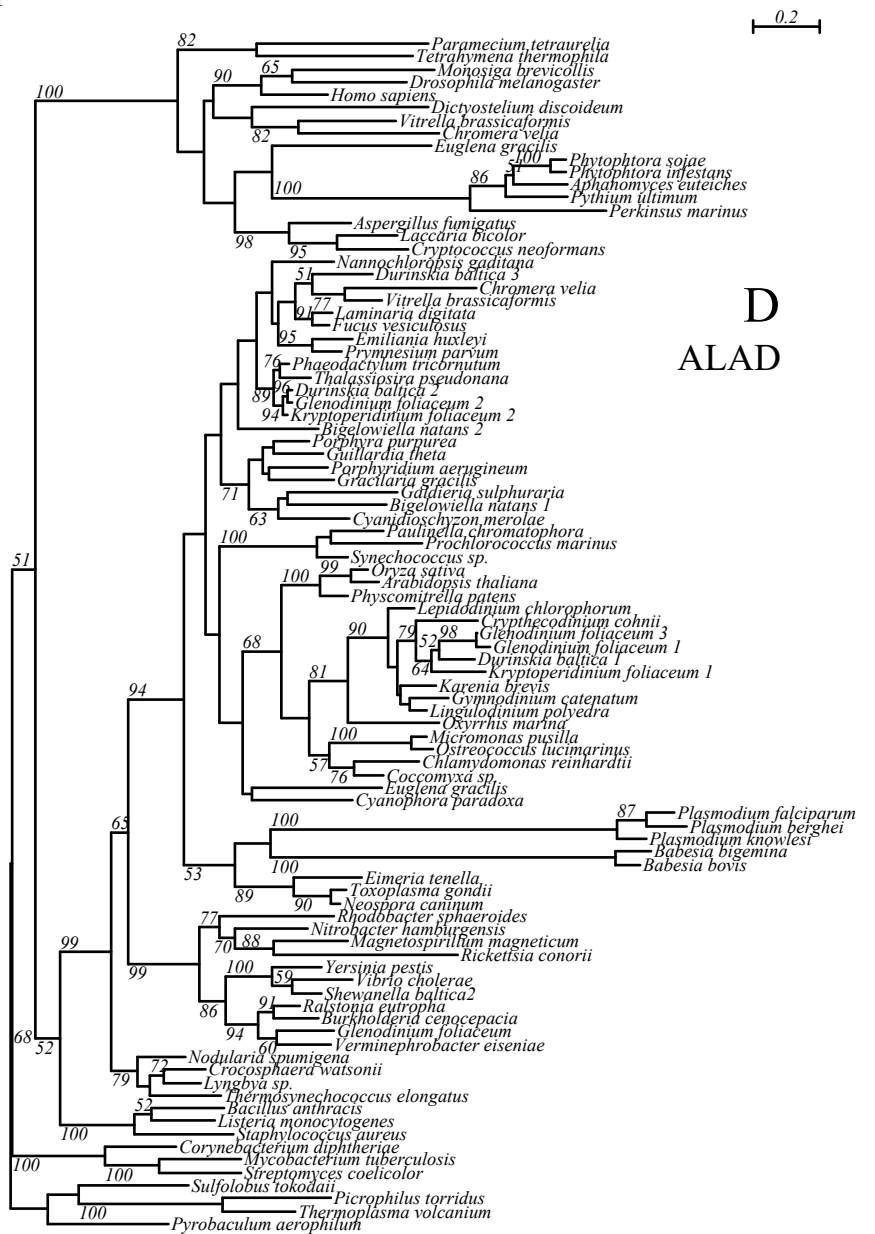

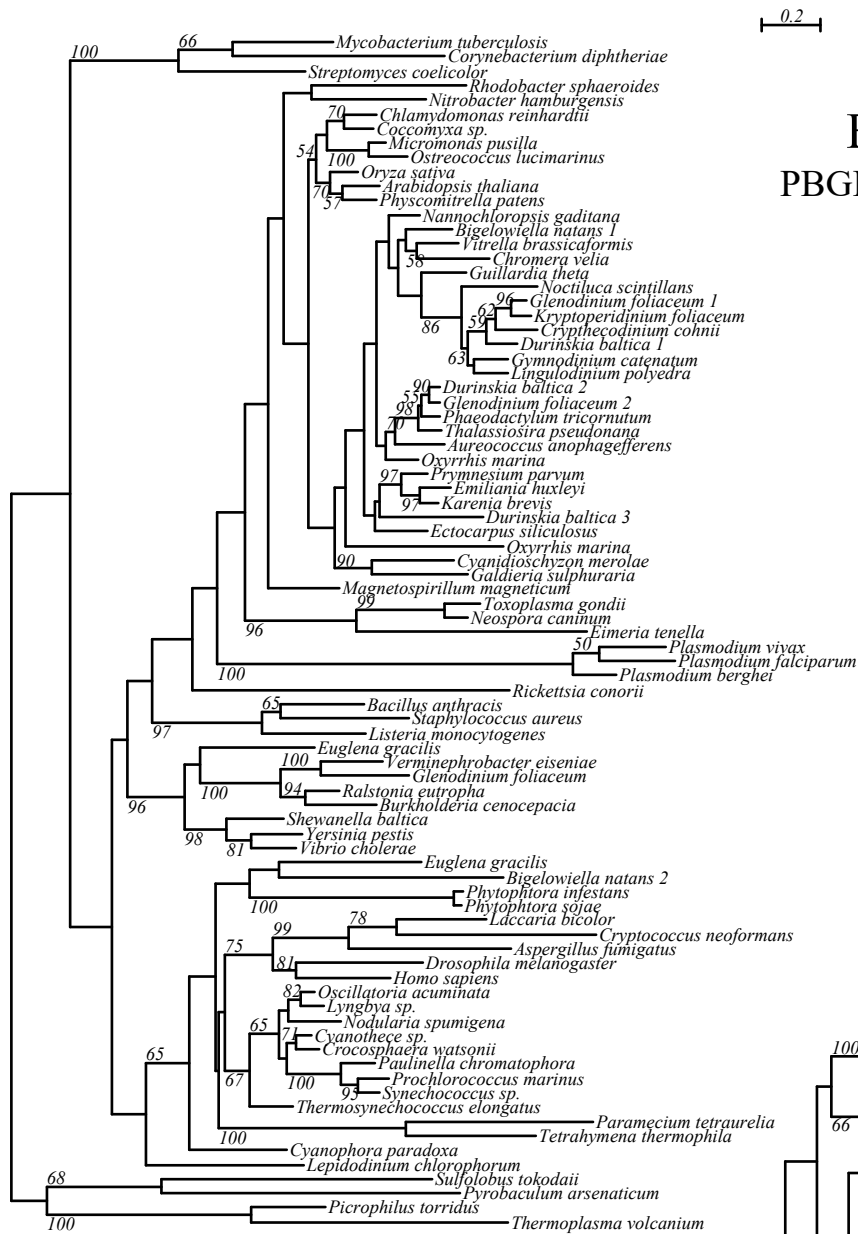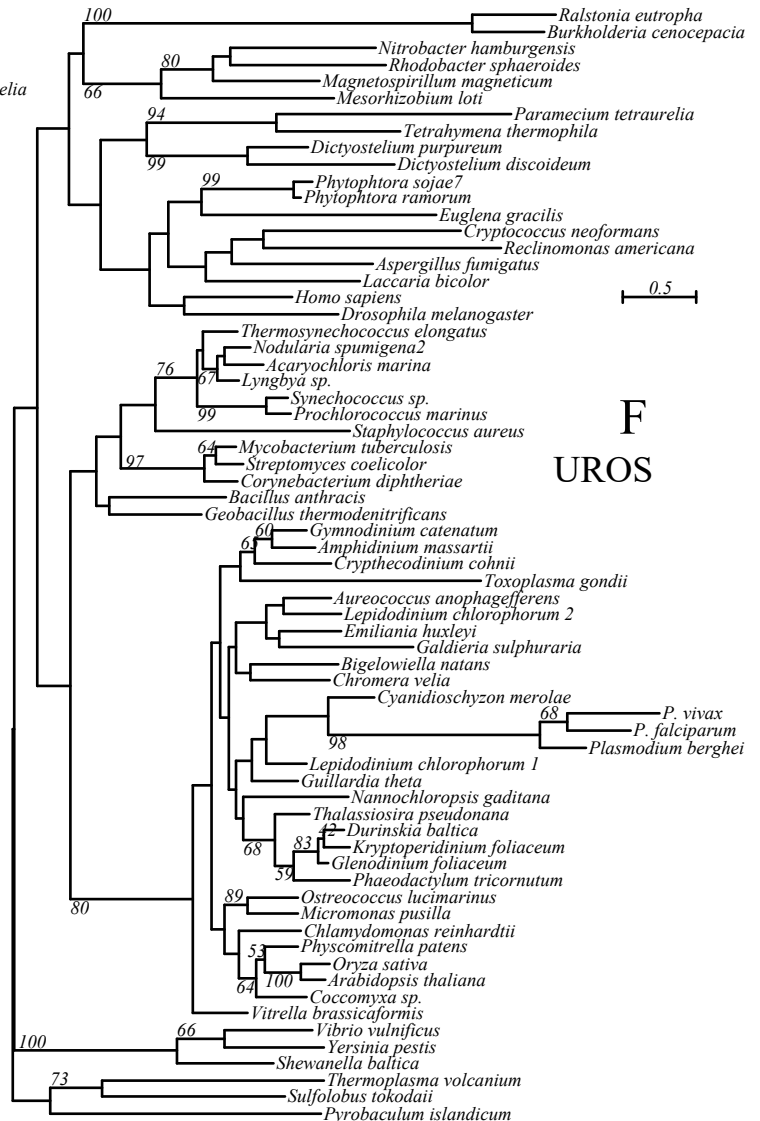

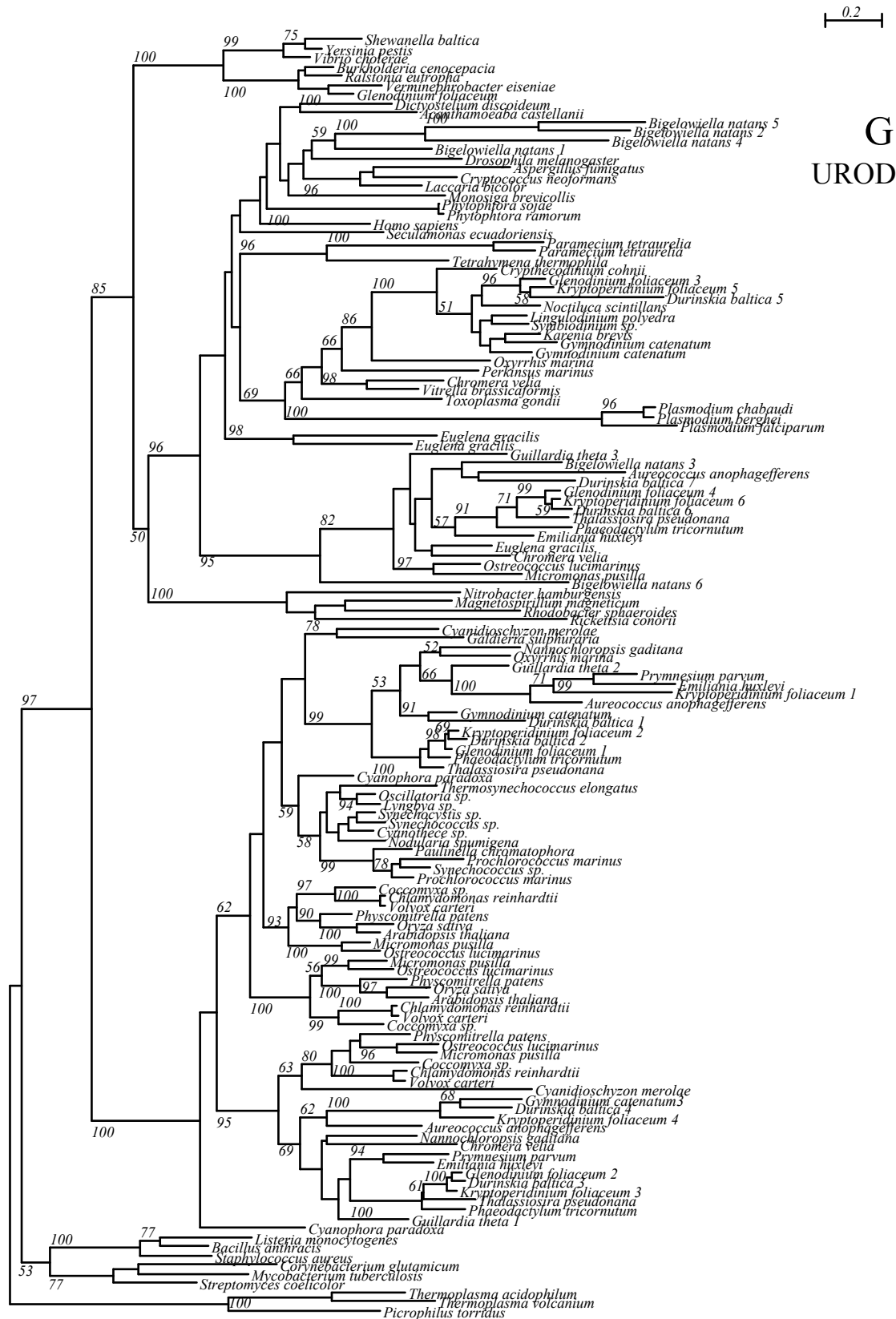

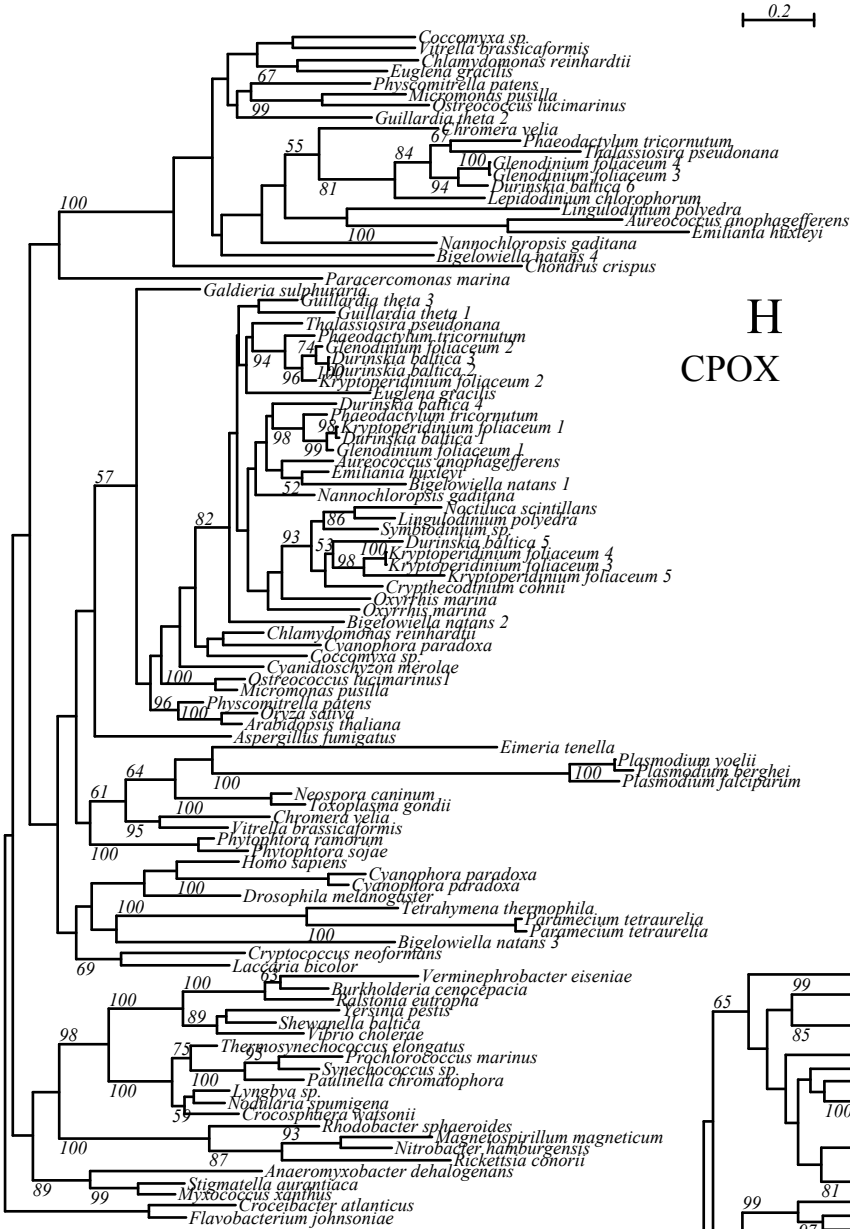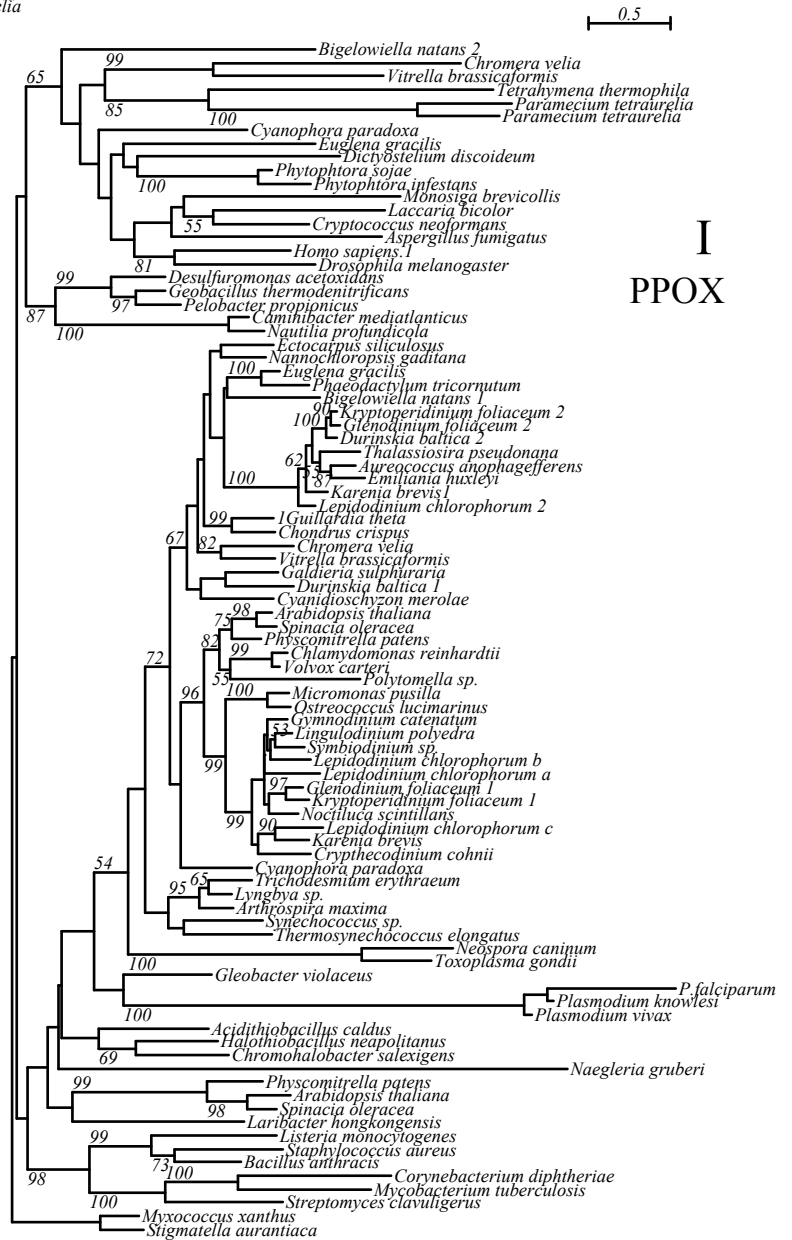

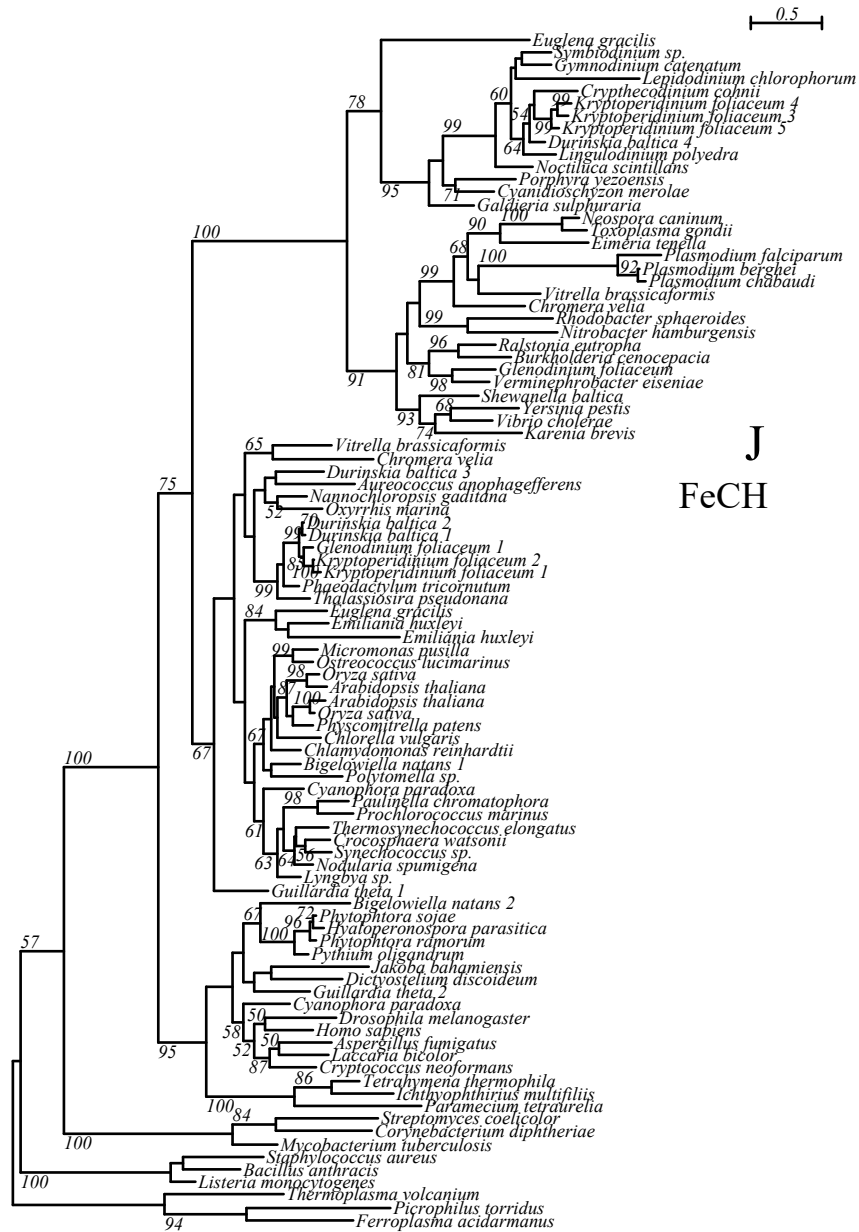

Supplement: S2 Fig — Numbers near branches indicate bootstrap values; only support values greater than 50 are shown. A, ALAS—delta-aminolevulinic acid synthase; B, GTR—glutamate-tRNA reductase; C, GSA—glutamate-1-semialdehyde aminotransferase; D, ALAD—aminolevulinic acid dehydratase; E, PBGD—porphobilinogen deaminase; F, UROS—uroporphyrinogen synthase; G, UROD—uroporphyrinogen decarboxylase; H, CPOX—coproporphyrinogen oxidase; I, PPOX—protoporphyrinogen oxidase; J, FeCH—ferrochelatase. LcPPOXa, -b, -c; LcUROSa, -b = non-overlapping protein models, putatively fragments of LcPPOX1 and LcUROS. (PDF) [file pone.0166338.s002.pdf]
