# Supplementary material for: Evolution of the Tetrapyrrole Biosynthetic Pathway in Secondary Algae: Conservation, Redundancy and Replacement
Source: PLoS One. 2016 Nov 18;11(11):e0166338. doi: 10.1371/journal.pone.0166338 (PMC5115734; doi:10.1371/journal.pone.0166338)
Supplement: S1 Table — Targeting probabilities were determined using SignalP and TargetP as described in Materials and Methods. Respective targeting peptide sequences are listed. The presence of a signal peptide followed by a chloroplast targeting peptide (cTP) implies localization to the plastid; mitochondrial enzymes encode mitochondrial targeting peptide presequences, while cytoplasmic enzymes lack presequences. If available, models with longer N-termini (e.g. Pasa, Fgenesh) were included in pre-sequence analysis and are listed in the table. (PDF) [file pone.0166338.s003.pdf]

**GTR**	SP probability	SP sequence	TP probability	TP sequence	Origin
**BnGTR** ***Bigna1|58152***	not predicted		cTP: 0.350 mTP: 0.565	MSGTSRTQPTSSSSNLIRLATT LPMSVREKLAIPEAEWKAQA AKLNQMPNIDEAGILSTCNRF EVYYVAHDTQAANEQLMSFL KEKSGLTDSELSPNLFFHDGY EATK	cyanobacterial (plastid)
**GtGTR** ***Guith1|160073***	1.000	MQLVAGLVLLSAASCAA	cTP: 0.214 mTP: 0.520	FSPVAPLRVVGSSAARLNVSP ARFA	cyanobacterial (plastid)

**GSA-AT**	SP probability	SP sequence	TP probability	TP sequence	Origin
**BnGSA** ***Bigna1|46248***	0.585	MQTHYTSFPSSSARSNKYL VLSLVANAILLVGIGCVSL	cTP: 0.569 mTP: 0.607	QGPLR	cyanobacterial (plastid)
**GtGSA** ***Guith1|144401***	0.998	MKRKWFLIFAFLSMRQLM A	cTP: 0.321 mTP: 0.400	FSPRPLPLPGSKRSLLKVCP SI	cyanobacterial (plastid)

**ALAS**	SP probability	SP sequence	TP probability	TP sequence	Origin
**BnALAS** ***Bigna1|41839***	not predicted		mTP: 0.133	not predictable	mitochondrial
Guillardia theta	doesn’t have ALAS				

**ALAD**	SP probability	SP sequence	TP probability	TP sequence	Origin
**BnALAD** ***Bigna1|56191***	not predicted		mTP: 0.857	MLRAILRRGRGFRRPCTGGT ILSRSPGRTF	cyanobacterial (plastid)
**BnALAD2** ***Bigna1|85530***	0.897	MNTNANLRCSLATNVALAI LFVTMAFMGQNTLSSA	cTP: 0.187 mTP: 0.732	LSVQRTVVSPAVTMRSGRM ASFAKVSAPE	cyanobacterial (plastid)
**GtALAD** ***Guith1|156959***	not predicted		cTP: 0.753	MKESIYGLKQRPNGDIWVPQ RARPRRNRKNEGLR	cyanobacterial (plastid)
**GtALAD** ***Guith1|156959*** **(Fgenesh ab initio model)**	0.999	MFVRRSLLLTVSCAVLAAT NA	cTP: 0.425 mTP: 0.700	FVAVQPSARL	cyanobacterial (plastid)

**PBGD**	SP probability	SP sequence	TP probability	TP sequence	Origin
**BnPBGD1** ***Bigna1|90804***	0.886	MRVQKFEASIALNIALGVVF LCMLHGRMG	cTP: 0.697	HQQVASAPVITRSVTSTPINY HAQRGLRRDLGMR	a-proteobacterial (mitochondrial)
**BnPBGD2** ***Bigna1|86774***	0.797	MDDRMLLPALATAGALA	not predicted		eukaryotic
**GtPBGD** ***Guith1|159565*** **(Pasa model)**	1.000	MLRALSAAACAAAMLAS ADA	cTP: 0.753	FLPSTPLSNAAKIRPATSRIST IMSGEPVIKLGTRGSPLAL	a-proteobacterial (mitochondrial)
**GtPBGD** ***Guith1|159565***	not predicted		cTP: 0.116 mTP: 0.461	MSGEPVIKLGTRGSPLALAQ AYETRRRL	a-proteobacterial (mitochondrial)

UROS	SP probability	SP sequence	TP probability	TP sequence	Origin
**BnUROS** ***Bigna1|38206*** **(Pasa model)**	0.996	LPTTPPCPTRAMFLLAAASLTALLALSRTWT	cTP: 0.155	SSFSSRRIGRGARLA	cyanobacterial (plastid)
**BnUROS** ***Bigna1|38206***	not predicted		not predicted		cyanobacterial (plastid)
**GtUROS** ***Guith1|70083*** **(Fgenesh ab initio model)**	0.999	MAMAMVLRVLAGLSLMAGGEA	cTP: 0.866	FVCYQVSSPHSPLASPSLCYQHSRCSLPPR	cyanobacterial (plastid)
**GtUROS** ***Guith1|70083***	not predicted		not predicted		cyanobacterial (plastid)

UROD	SP probability	SP sequence	TP probability	TP sequence	Origin
**BnUROD1** ***Bigna1|42002***	not predicted		not predicted		eukaryotic
**BnUROD2** ***Bigna1|45626***	not predicted		not predicted		eukaryotic
**BnUROD3** ***Bigna1|92414***	not predicted		mTP: 0.567	MILFQAPQHDLLLRAARG	primary host nucleus
**BnUROD4** ***Bigna1|85794***	not predicted		cTP: 0.126 mTP: 0.764	MALTCNLPRSRSRSSRSRRSHHSLPAL	eukaryotic
**BnUROD5** ***Bigna1|84362***	0.995	MLFLLLVTTALASPTST	cTP: 0.876	IKSVASNSLNGLRGAEFQGIRSLSRGALQQLARSRRANRQSSRRLGERRRVSASAENFPALKNDLLIR	eukaryotic

**BnUROD6** ***Bigna1|68242***	1.000	MATVVLVIVSLTALAMLP PTLS	cTP: 0.078 mTP: 0.931	SVGVRL	primary host nucleus
**GtUROD1** ***Guith1|159098***	0.933	MAAAWTAAALLSKASRA		not predicted	cyanobacterial (plastid)
**GtUROD2** ***Guith1|154354***	0.999	MFLLRSAAVASFLLACGL PYSSA	cTP: 0.570	FVGSGLTVTPKDARLRTAV SATASRSTPLGLKMEDPLLL R	cyanobacterial (plastid)
**GtUROD3** ***Guith1|76641***	0.999	MVMAVGAVIAVALAMA AEVQG	cTP: 0.902	YAMTRAGGANFVSGLSTFS SSSSLTGLPLSSRACR	primary host nucleus

**CPOX**	SP probability	SP sequence	TP probability	TP sequence	Origin
**BnCPOX1** ***Bigna1| 47005*** **(Pasa model)**	0.997	MMSPASLVGLLALFAV SMLVATGPA	cTP: 0.316 mTP: 0.834	TRGHFQVARSAPARS	eukaryotic/primary host nucleus
**BnCPOX1** ***Bigna1|47005***	not predicted		not predicted		eukaryotic/primary host nucleus
**BnCPOX2** ***Bigna1|47366*** **(Pasa model)**	not predicted		cTP: 0.214	MIGWFCFSLLAGDVQGLSRM QIISRERDPLVR	eukaryotic/primary host nucleus
**BnCPOX2** ***Bigna1|47366***	not predicted		not predicted		eukaryotic/primary host nucleus
**BnCPOX3** ***Bigna1|91492***	not predicted		not predicted		eukaryotic/secondary host nucleus
**BnCPOX4** ***Bigna1|44533*** **(Pasa model)**	0.999	FGSGSAQSGTEMLLLAPLW LTMASTLA	cTP: 0.678 mTP: 0.718	ASARGVEAGARY	uncertain origin
**BnCPOX4** ***Bigna1|44533***	not predicted		not predicted		uncertain origin

**GtCPOX1** ***Guith1|88140***	0.982	MFMRSFLIATTMTMVVNP VSG	cTP: 0.269 mTP: 0.414	FLPSSLPSFRVSRSNLAPAAIS TRM	primary host nucleus
**GtCPOX2** ***Guith1|157905*** **(Pasa model)**	1.000	MLVPCRMSGAAALLPLAL LAQAALSSA	cTP: 0.886	YVTSPSSLSGIRTNFASSVSKS RRAYPLSWARALRA	primary host nucleus
**GtCPOX2** ***Guith1|157905***	not predicted		not predicted		primary host nucleus
**GtCPOX3** ***Guith1|164206***	not predicted		not predicted		uncertain origin in primary alga

**PPOX**	SP probability	SP sequence	TP probability	TP sequence	Origin
**BnPPOX1** ***(Bigna1|56780)***	not predicted but *	*possesses an N-terminal extension	not predicted		cyanobacterial (plastid)
**BnPPOX2** ***(Bigna1|85182)***	not predicted		mTP: 0.775	MASGKSLRVAVVGGGISGLS AAFHLQRGLPKGSTLKLIEAS DRVGGWIHSKRKGEFLFECG PATLRG	eukaryotic
**BnPPOX2** ***(Bigna1|85182)***	0.878	MASGKSLRVAVVGGGISGLS AA	not predicted		eukaryotic
**GtPPOX** ***Guith1|98119*** **(Pasa model)**	0.983	MAMSRQVLLMVTMAAVRT KA	cTP: 0.824	WVTSPSMLSSPALLRMPSCS WTGKSSVRGARDSSVQFRG RSARFGAGV	cyanobacterial (plastid)
**GtPPOX** ***Guith1|98119***	0.661	MVTMAAVRTKAWVTSPS MLSSPALLRMPSCSWT	cTP: 0.104 mTP: 0.551	GKSSVRG	cyanobacterial (plastid)

**FeCH**	SP probability	SP sequence	TP probability	TP sequence	Origin
**BnFeCH1** ***(Bigna1|134648)***	0.984	MDLKHRGRGWLAVWAIAF VFLPHVAST	cTP: 0.887	SRLASTSAPRMPPSISKTPF ARRLFCKVQECGGRLQAS KTPIPRRLR	cyanobacterial (plastid)
**BnFeCH2** ***(Bigna1|125872)***	not predicted		mTP: 0.971	MLSFRHAWKSSRGARLLA RWK	eukaryotic
**GtFeCH1** ***(Guith1|83978 Guith1|132095)***	0.986	MLRASCRWLSLAICFTAVSS	cTP: 0.429<br>mTP: 0.875	FQHAPSLVLRSTRSPALSR RGSVSSARMA	cyanobacterial (plastid)
**GtFeCH2** ***(Guith1|157086)*** **minus 36AA**	not predicted		mTP: 0.842	MQLPMQKMLGSWIARRRS PKIMKQYAEIGGGSPIGKW TEIQGKKLERHLDEMCPET APHKTYIAFRY	eukaryotic
**GtFeCH2** ***(Guith1|157086)***	not predicted		not predicted		eukaryotic
