# Supplementary material for: Evolution of the Tetrapyrrole Biosynthetic Pathway in Secondary Algae: Conservation, Redundancy and Replacement
Source: PLoS One. 2016 Nov 18;11(11):e0166338. doi: 10.1371/journal.pone.0166338 (PMC5115734; doi:10.1371/journal.pone.0166338)
Supplement: S2 Table — Number of reads and bases of two libraries are listed after quality trimming (two reads per library, see Material and Methods). The resulting number of contigs and coding sequences were analyzed using the BUSCO pipeline with a set of 429 BUSCO groups of orthologs. Ortholog counts: C = complete; D = duplicated; F = fragments; M = missing. (PDF) [file pone.0166338.s004.pdf]

	Reads/bases after Trimming (>96% of input)	Contigs/ LongORFs	BUSCO summary
Ld-mR	8,732,015/1,689,227,046	153854/148639	C:150[D:59],F:109,M:170(39%)
	8,645,235/1,620,283,360		
Ld-MN	12,747,584/2,776,204,806	184793/211602	C:316[D:240],F:71,M:42(9.7%)
	12,290,654/2,531,281,646		
