# Supplementary material for: Evolution of the Tetrapyrrole Biosynthetic Pathway in Secondary Algae: Conservation, Redundancy and Replacement
Source: PLoS One. 2016 Nov 18;11(11):e0166338. doi: 10.1371/journal.pone.0166338 (PMC5115734; doi:10.1371/journal.pone.0166338)
Supplement: S3 Table — Gene copy designation for species of interest in this study is shown in brackets according to their designation in respective trees (an asterisk marks putative contaminant sequences). Databases: CGP = Cyanophora Genome Project; CryptoDB = Cryptosporidium Genomic Resource; gb = GenBank; Gruber ea. = Gruber et al. 2015 Plant J, 10.1111/tpj.12734; jgi = DOE Joint Genome Institute; MMETSP = Marine Microbial Eukaryote Transcriptome Sequencing Project; Nori = NoriBLAST, Porphyra Genome Project; psb = bioinformatics.psb.ugent.be; VH = Courtesy of Vladimír Hampl, unpublished; Cmb = combined samples. (PDF) [file pone.0166338.s005.pdf]

Species	Database	Sample Name	Sequence ID
**delta-aminolevulinic acid synthase (ALAS):**			
Acanthamoeba castellani	HGSC		Contig287, Contig1624
Agrobacterium tumefaciens	gb		ABD39319
Amycolatopsis orientalis	gb		WP_016333295.1
Anaplasma phagocytophilum	gb		YP_505769
Aspergillus fumigatus	gb		XP_754006
Aurantimonas sp.	gb		ZP_01228314
Azorhizobium caulinodans	gb		WP_012168865.1
Beijerinckia indica	gb		WP_041778950.1
Bigelowiella natans	jgi		Bigna1|41839
Bordetella petrii	gb		CAP41660.1
Bradyrhizobium sp.	gb		YP_001239995
Brucella melitensis	gb		NP_540521
Caulobacter sp.	gb		YP_001684674
Cryptococcus neoformans	gb		XP_569249
Dictyostelium discoideum	gb		XP_641014
Dinoroseobacter shibae	gb		WP_012180202.1
Drosophila melanogaster	gb		NP_477281
Ehrlichia canis	gb		YP_302705
Erythrobacter sp.	gb		ZP_01039424
Euglena gracilis	gb		AF325915, EC678670, EC675939, EC674381, EL580553, EC681893
Fulvimarina pelagi	gb		ZP_01437352
Gluconobacter oxydans	gb		YP_192033
Hartmannella vermiformis	gb		EC134460
Hoeflea phototrophica	gb		ZP_02167528
Homo sapiens	gb		NP_000679
Hyphomonas neptunium	gb		YP_760760
Chromobacterium violaceum	gb		NP_900473
Jannaschia sp.	gb		YP_509875
Laccaria bicolor	gb		XP_001874728
Magnetospirillum gryphiswaldense	gb		CAM76191.1
Maricaulis maris	gb		YP_756770
Methylobacterium radiotolerans	gb		KTS48499.1

Mesorhizobium loti	gb		NP_107074
Monosiga brevicollis	gb		XP_001749025
Mycosphaerella graminicola	jgi		Mycgr3|105601
Nematostella vectensis	gb		XP_001638269
Neorickettsia sennetsu	gb		YP_506696
Neospora caninum	gb		XP_003883025
Nitrobacter winogradskyi	gb		YP_316862
Novosphingobium aromaticivorans	gb		YP_497071
Oceanibulbus indolifex	gb		EDQ04014
Oceanicaulis sp.	gb		EAP90895.1
Oceanicola granulosus	gb		ZP_01156180
Ochrobactrum anthropi	gb		WP_012090709.1
Oligotropha carboxidovorans	gb		ZP_03158741
Orientia tsutsugamushi	gb		WP_047220874.1
Paracoccus denitrificans	gb		ZP_00629563
Paramecium tetraurelia	gb		XP_001433163
Parvibaculum lavamentivorans	gb		WP_041536776.1
Parvularcula bermudensis	gb		ZP_01016873
Perkinsus marinus	gb		XP_002772547
Phycomyces blakesleeanus	jgi		Phybl2|78290
Phytophthora ramorum	jgi		Physo2|248728
Phytophthora sojae	jgi		Physo1_1|143415
Plasmodium falciparum	gb		XP_001350846
Plasmodium knowlesi	gb		CAQ42411
Plasmodium yoelii	gb		XP_725921
Pythium ultimum	gb		FF054624, EL775879, EL776881
Roseovarius sp.	gb		ZP_01034511
Rhodobacter capsulatus	gb		KQB15076.1
Rhodobacter sphaeroides	gb		YP_354544
Rhizobium etli	gb		YP_471320
Rhodoblastus acidophilus	gb		ABB90620
Rhodopseudomonas palustris	gb		YP_531206
Rhodospirillum rubrum	gb		YP_427595
Rickettsia conorii	gb		NP_360940

Roseobacter denitrificans	gb		YP_683041
Sagittula stellata	gb		WP_005858062.1
Seculamonas ecuadoriensis	gb		EC817129
Silicibacter sp.	gb		EEW57342.1
Sphingomonas sp.	gb		ZP_01304166
Sphingopyxis alaskensis	gb		WP_011541507.1
Streptomyces aizunensis	gb		AAX98209
Streptomyces ghanaensis	gb		ABJ90148
Streptomyces nodosus	gb		AAO62615
Sulfitobacter sp.	gb		ZP_00964108
Tetrahymena thermophila	gb		XP_001030813
Toxoplasma gondii	gb		XP_002365083
Trichoplax adhaerens	jgi		Triad1|55298
Wolbachia endosymbiont of Brugia malayi	gb		YP_197966
Xanthobacter autotrophicus	gb		YP_001417027
Zymomonas mobilis	gb		YP_162933

glutamate-tRNA reductase (GTR):

Bigelowiella natans	jgi		Bigna1|58152
Crypthecodinium cohnii WH (Provasly/Seligo)	MMETSP	Cmb:P0323-26	PEP_0193872364
Cyanophora paradoxa	CGP		NP_043163
Durinskia baltica CSIRO CS-38	MMETSP	Cmb:P0116-17	Light: 6735_1 (1), PEP_0199916038 (2), Dark: 45651_1 (3)
Glenodinium foliaceum CCAP 1116/3	MMETSP	Cmb:P0118-19	Light: 42925_1 (1), 25863_1 (2), PEP_0188358838 (3), PEP_0188432100 (*)
Guillardia theta	jgi		Guith1|160073
Gymnodinium catenatum GC744	MMETSP	P0784	PEP_0117584166, PEP_0117466520, PEP_0117512884, PEP_0117479456
Chondrus crispus	gb		XP_005709872.1
Karenia brevis SP1	MMETSP	Cmb:P0573-74	PEP_0189026816
Kryptoperidinium foliaceum CCMP 1326	MMETSP	Cmb:P0120-21	PEP_0189671012
Lepidodinium chlorophorum	gb		KX344037
Lingulodinium polyedra CCMP 1738	MMETSP	Cmb:P1032-35	PEP_0190023468, PEP_0189963080
Noctiluca scintillans	MMETSP	P0253	PEP_0194537388
Oxyrrhis marina	MMETSP	Cmb:P0468-71	PEP_0190339716
*Symbiodinium sp.*CCMP421	MMETSP	P1110	PEP_0181502172

glutamate-1-semialdehyde aminotransferase (GSA):

Alexandrium ostenfeldii	gb		HO656134
Aureococcus anophagefferens	gb		EGB05641
Bigelowiella natans	jgi		Bigna1|46248
Bradyrhizobium sp.	gb		YP_001207580
Calothrix sp.	gb		WP_019490327
Crypthecodinium cohnii WH (Provasly/Seligo)	MMETSP	Cmb:P0323-26	PEP_0193849852, PEP_0193936778, PEP_0193930080
Curvibacter lanceolatus	gb		WP_019576179
Cyanophora paradoxa	CGP		ConsensusfromContig8081
Cycloclasticus sp.	gb		WP_016390789
Durinskia baltica CSIRO CS-38	MMETSP	Cmb:P0116-17	Light: 57900_1 (2), 47532_1 (6), 43571_1 (7), Dark: 15683_1 (4), 4094_1 (8), PEP_0199998778 (1), PEP_0199978498 (3), PEP_0200062182 (5), PEP_0199929374 (9), PEP_0200079780 (10)
Emiliania huxleyi	gb		XP_005785235
Glenodinium foliaceum CCAP 1116/3	MMETSP	Cmb:P0118-19	PEP_0188235580 (1), PEP_0188246880 (2), Light: 25514_1 (3), 49641_1 (4), 44973_1 (5), 11403_1 (7), PEP_0188430050 (6), PEP_0188239508 (8), Dark: 2970_1 (*)
Guillardia theta	MMETSP	P0046	PEP_0188235580
Gymnodinium catenatum GC744	MMETSP	P0784	PEP_0117512748, PEP_0117594084, PEP_0117560678
Hepalosiphon sp.	gb		WP_053455278
Karenia brevis SP1	MMETSP	Cmb:P0573-74	PEP_0189085658, PEP_0189017670, PEP_0189088064
Kryptoperidinium foliaceum CCMP 1326	MMETSP	Cmb:P0120-21	Light: 31046_1 (1), 88664_1 (2), 39526_1 (3), 27516_1 (8), 84022_1 (11), Dark:75325_1 (4), 72963_1 (5), 50699_1 (6), 45695_1 (7), 24126_1 (9), PEP_0189925348 (10), PEP_0189720460 (12)
Lepidodinium chlorophorum	gb		KX344033 (1), KX344034 (2), KX344035 (3), KX344036 (4)
Leptothrix cholodnii	gb		YP_006557962
Lingulodinium polyedra CCMP 1738	MMETSP	Cmb:P1032-35	PEP_0189998874, PEP_0189975894, PEP_0190087520
Marinobacter sp.	gb		YP_006557962
Mastigocoleus testarum	gb		WP_027845568
Micromonas sp.	gb		XP_002508168
Nitrospira moscoviensis	gb		WP_053381824
Noctiluca scintillans	MMETSP	P0253	PEP_0194528010, PEP_0194503378, PEP_0194503836
Paulinella chromatophora	gb		YP_002048687

Pelomonas sp.	gb		WP_056194614, WP_056875017
Pyrocystis lunula	gb		BU582413
Selaginella moellendorffii	gb		XP_002987521, XP_002987738
Symbiodinium A1	gb		GA418227
*Symbiodinium sp.*CCMP421	MMETSP	P1110	PEP_0181426964, PEP_0181524020

aminolevulinic acid dehydratase (ALAD):

Bigelowiella natans	jgi		Bigna1|56191 (1), Bigna1|85530 (2)
Crypthecodinium cohnii WH (Provasly/Seligo)	MMETSP	Cmb:P0323-26	PEP_0193843234
Cyanophora paradoxa	CGP		ConsensusfromContig54939
Durinskia baltica CSIRO CS-38	MMETSP	Cmb:P0116-17	Light: 3134_1 (1), PEP_0199922310 (2), PEP_0200064228 (3)
Glenodinium foliaceum CCAP 1116/3	MMETSP	Cmb:P0118-19	PEP_0188249364 (1), PEP_0188432888 (2), Dark: 41336_1 (3), PEP_0188280572 (*)
Gracilaria gracilis	gb		CAC36153
Guillardia theta	jgi		Guith1|156959
Gymnodinium catenatum GC744	MMETSP	P0784	PEP_0117519910
Chromera velia	CryptoDB		Cvel_108, Cvel_13826
Karenia brevis SP1	MMETSP	Cmb:P0573-74	PEP_0189092812
Kryptoperidinium foliaceum CCMP 1326	MMETSP	Cmb:P0120-21	Light: 6107_1 (1), 40815_1 (2)
Lepidodinium chlorophorum	gb		KX344038
Lingulodinium polyedra CCMP 1738	MMETSP	Cmb:P1032-35	PEP_0190001018
Nannochloropsis gaditana	gb		EWM27133.1
Oxyrrhis marina	MMETSP	Cmb:P0468-71	PEP_0190347748
Paulinella chromatophora	gb		YP_002049307
Phaeodactylum tricornutum	gb		XP_002177697
Porphyra purpurea	Nori		P_purpurea_esisotig05989
Porphyridium aerugineum	MMETSP		PEP_0184706278
Vitrella brassicaformis	CryptoDB		Vbra_4124, Vbra_4750

porphobilinogen deaminase (PBGD):

Bigelowiella natans	jgi		Bigna1|90804 (1), Bigna1|86774 (2)
Crypthecodinium cohnii WH (Provasly/Seligo)	MMETSP	Cmb:P0323-26	PEP_0193852140
Cyanophora paradoxa	CGP		ConsensusfromContig7377
Cyanothece sp.	gb		WP_012595314

Durinskia baltica CSIRO CS-38	MMETSP	Cmb:P0116-17	PEP_0199945040 (1), PEP_0199919972 (2), PEP_0199940260 (3)
Ectocarpus siliculosus	psb		ES0172G00250
Eimeria tenella	gb		CDJ44721.1
Galdieria sulphuraria	gb		XP_005704560.1
Glenodinium foliaceum CCAP 1116/3	MMETSP	Cmb:P0118-19	PEP_0188260592 (1), PEP_0188362918 (2), Dark: 71221_1 (*)
Guillardia theta	jgi		Guith1|159565
Gymnodinium catenatum GC744	MMETSP	P0784	PEP_0117526388
Chromera velia	CryptoDB		Cvel_26028
Kamptonema	gb		WP_007355703
Karenia brevis SP1	MMETSP	Cmb:P0573-74	PEP_0189092548
Kryptoperidinium foliaceum CCMP 1326	MMETSP	Cmb:P0120-21	Light: 24070_1
Lepidodinium chlorophorum	gb		KX344039
Lingulodinium polyedra CCMP 1738	MMETSP	Cmb:P1032-35	PEP_0190014442
Nannochloropsis gaditana	gb		EWM30321.1
Noctiluca scintillans	MMETSP	P0253	PEP_0194516312
Oxyrrhis marina	MMETSP	Cmb:P0468-71	PEP_0190367812
Paulinella chromatophora	gb		YP_002049482
Phaeodactylum tricornutum	gb		XP_002179459
Prymnesium parvum Texoma1	MMETSP	Cmb:P0006-8, P0814-15	PEP_0191201180
Vitrella brassicaformis	CryptoDB		Vbra_10189

uroporphyrinogen synthase (UROS):

Amphidinium massartii CS-259	MMETSP	P0689_2	PEP_0178420724
Bigelowiella natans	jgi		Bigna1|228454
Crypthecodinium cohnii WH (Provasly/Seligo)	MMETSP	Cmb:P0323-26	PEP_0193844694
Durinskia baltica CSIRO CS-38	MMETSP	Cmb:P0116-17	PEP_0199908334
Glenodinium foliaceum CCAP 1116/3	MMETSP	Cmb:P0118-19	Light: 23567_1
Guillardia theta	jgi		Guith1|70083
Gymnodinium catenatum GC744	MMETSP	P0784	PEP_0117540008
Chromera velia	CryptoDB		Cvel_15018
Kryptoperidinium foliaceum CCMP 1326	MMETSP	Cmb:P0120-21	PEP_0189819568
Lepidodinium chlorophorum	gb		KX344040 (a), KX344041 (b)
Nannochloropsis gaditana	gb		EWM24015.1

Phaeodactylum tricornutum	gb		XP_002178352
Vitrella brassicaformis	CryptoDB		Vbra_17271

uroporphyrinogen decarboxylase (UROD):

Bigelowiella natans	jgi		Bigna1|42002 (1), Bigna1|45626 (2), Bigna1|92414 (3), Bigna1|85794 (4), Bigna1|84362 (5), Bigna1|68242 (6)
Coccomyxa subellipsoidea	gb		XP_005651296, XP_005647200, XP_005651957
Crypthecodinium cohnii WH (Provasly/Seligo)	MMETSP	Cmb:P0323-26	PEP_0193896856
Cyanophora paradoxa	CGP		ConsensusfromContig53895, ConsensusfromContig7761
Cyanothece sp.	gb		WP_015954684
Durinskia baltica CSIRO CS-38	MMETSP	Cmb:P0116-17	PEP_0200045368 (1),PEP_0199917834 (2), Light: 4680_1 (3), 37008_1 (4), 41392_1 (5), 5737_1 (6), 64742_1 (7)
Euglena gracilis	VH		N/A
Galdieria sulphuraria	gb		XP_005703762.1
Glenodinium foliaceum CCAP 1116/3	MMETSP	Cmb:P0118-19	PEP_0188359110 (1), Light: 26329_1 (2), 2986_1 (3), 25789_1 (4), Dark: 5272_1 (*)
Guillardia theta	jgi		Guith1|159098 (1), Guith1|154354 (2), Guith1|76641 (3)
Gymnodinium catenatum GC744	MMETSP	P0784	PEP_0117476542, PEP_0117544934, PEP_0117571066, PEP_0117527892
Chromera velia	CryptoDB		Cvel_31936, Cvel_14720, Cvel_5098
Kamptonema			WP_007355896
Karenia brevis SP1	MMETSP	Cmb:P0573-74	PEP_0189097350
Kryptoperidinium foliaceum CCMP 1326	MMETSP	Cmb:P0120-21	Light: 3210_1 (1), 12605_1 (2), 29038_1 (3), 7294_1 (4), 68159_1 (6), PEP_0189714812 (5)
Lingulodinium polyedra CCMP 1738	MMETSP	Cmb:P1032-35	PEP_0190035308
Nannochloropsis gaditana	gb		EWM30174.1, EWM29859.1
Noctiluca scintillans	MMETSP	P0253	PEP_0194508272
Oxyrrhis marina	MMETSP	Cmb:P0468-71	PEP_0190314344, PEP_0190323088
Paulinella chromatophora	gb		YP_002049486
Phaeodactylum tricornutum	gb		XP_002178653, XP_002180946, XP_002184319
Prochlorococcus marinus	gb		WP_011818087, WP_011125231
Prymnesium parvum Texoma1	MMETSP	Cmb:P0006-8, P0814-15	PEP_0191238938, PEP_0191228900
*Symbiodinium sp.*CCMP421	MMETSP	P1110	PEP_0181406244
Synechococcus sp.	gb		WP_012306451

Synechocystis sp.	gb		WP_010874048
Vitrella brassicaformis	CryptoDB		Vbra_4036

coproporphyrinogen oxidase (CPOX):

Bigelowiella natans	jgi		Bigna1|217286 (1), Bigna1|233393 (2), Bigna1|91492 (3), Bigna1|225296 (4)
Crypthecodinium cohnii WH (Provasly/Seligo)	MMETSP	Cmb:P0323-26	PEP_0193893022
Cyanophora paradoxa	CGP		ConsensusfromContig10670, ConsensusfromContig10833, ConsensusfromContig12168
Durinskia baltica CSIRO CS-38	MMETSP	Cmb:P0116-17	PEP_0199979854 (1), PEP_0199909788 (2), Dark: 18222_1 (3), PEP_0199945688 (4), PEP_0200057696 (5), PEP_0199911548 (6),
Glenodinium foliaceum CCAP 1116/3	MMETSP	Cmb:P0118-19	PEP_0188251708 (1), PEP_0188357876 (2), PEP_0188330552 (3), Light: 67739_1 (4)
Guillardia theta	jgi		Guith1|88140 (1), Guith1|87233 (2), Guith1|164330 (3)
Chondrus crispus	gb		XP_005714213.1
Chromera velia	CryptoDB		Cvel_2641, Cvel_21486
Kryptoperidinium foliaceum CCMP 1326	MMETSP	Cmb:P0120-21	Light: 85374_1 (1), 38472_1 (2), 27026_1 (4), PEP_0189813030 (3), Dark: 5047_1 (5)
Lepidodinium chlorophorum	gb		KX344042
Lingulodinium polyedra CCMP 1738	MMETSP	Cmb:P1032-35	PEP_0189985272, PEP_0189998628
Nannochloropsis gaditana	gb		EWM25951.1, EWM26442.1
Noctiluca scintillans	MMETSP	P0253	PEP_0194530262
Oxyrrhis marina	MMETSP	Cmb:P0468-71	PEP_0190301822, PEP_0190368530
Paracercomonas marina	gb		EW706343
Paulinella chromatophora	gb		YP_002049255
Phaeodactylum tricornutum	gb		XP_002186510, XP_002182874, XP_002179603
Phytophtora sojae	gb		XP_009532964, XP_009532965
*Symbiodinium sp.*CCMP421	MMETSP	P1110	PEP_0181433514
Vitrella brassicaformis	CryptoDB		Vbra_16068, Vbra_3027

protoporphyrinogen oxidase (PPOX):

Bigelowiella natans	jgi		Bigna1|56780 (1), Bigna1|85182 (2)
Crypthecodinium cohnii WH (Provasly/Seligo)	MMETSP	Cmb:P0323-26	PEP_0193880512
Cyanophora paradoxa	CGP		ConsensusfromContig53630, ConsensusfromContig7466

Durinskia baltica CSIRO CS-38	MMETSP	Cmb:P0116-17	Dark: 47539_1 (1) , PEP_0199927794 (2)
Euglena gracilis	VH		N/A
Glenodinium foliaceum CCAP 1116/3	MMETSP	Cmb:P0118-19	Light: 37425_1 (1), PEP_0188231826 (2)
Gleobacter violaceus	gb		WP_011140945
Guillardia theta	jgi		Guith1|98119
Gymnodinium catenatum GC744	MMETSP	P0784	PEP_0117558894
Homo sapiens	gb		NP_000300
Chondrus crispus	gb		XP_005718212.1
Chromera velia	CryptoDB		Cvel_18037
Karenia brevis SP1	MMETSP	Cmb:P0573-74	PEP_0189135782, PEP_0189009568
Kryptoperidinium foliaceum CCMP 1326	MMETSP	Cmb:P0120-21	PEP_0189916268 (1), Light: 13192_1 (2)
Lepidodinium chlorophorum	gb		KX344043 (a), KX344044 (b), KX344045 (c), KX344046 (2)
Lingulodinium polyedra CCMP 1738	MMETSP	Cmb:P1032-35	PEP_0189980810
Naegleria gruberi	gb		XP_002676536
Nannochloropsis gaditana	gb		XP_005854747.1
Noctiluca scintillans	MMETSP	P0253	PEP_0194536960
Phaeodactylum tricornutum	Gruber *ea.*		Phatr2a_50354
*Symbiodinium sp.*CCMP421	MMETSP	P1110	PEP_0181420296
Synechococcus sp.	gb		WP_011429973
Vitrella brassicaformis	CryptoDB		Vbra_22920

ferrochelatase:

Bigelowiella natans	jgi		Bigna1|134648 (1), Bigna1|224475 (2)
Crypthecodinium cohnii WH (Provasly/Seligo)	MMETSP	Cmb:P0323-26	PEP_0193842588
Cyanophora paradoxa	CGP		ConsensusfromContig26964, ConsensusfromContig38681
Durinskia baltica CSIRO CS-38	MMETSP	Cmb:P0116-17	Light: 5381_1 (1), 3444_1 (2), PEP_0200058834 (3), PEP_0199992624 (4)
Emiliania huxleyi	gb		XP_005784633, XP_005762401
Glenodinium foliaceum CCAP 1116/3	MMETSP	Cmb:P0118-19	PEP_0188265132 (1), PEP_0188435804 (*)
Guillardia theta	jgi		Guith1|132095 (1), Guith1|157086 (2)
Gymnodinium catenatum GC744	MMETSP	P0784	PEP_0117464132
Coccomyxa subellipsoidea	gb		XP_005644614
Chromera velia	CryptoDB		Cvel_18167, Cvel_26873
Karenia brevis SP1	MMETSP	Cmb:P0573-74	PEP_0189174344

Kryptoperidinium foliaceum CCMP 1326	MMETSP	Cmb:P0120-21	Light: 72684_1 (1), 14704_1 (2), PEP_0189920362 (3),<br>Dark: 30289_1 (4), PEP_0189771678 (5)
Lepidodinium chlorophorum	gb		KX344047
Lingulodinium polyedra CCMP 1738	MMETSP	Cmb:P1032-35	PEP_0190016732
Nannochloropsis gaditana			EWM27178.1
Noctiluca scintillans	MMETSP	P0253	PEP_0194512194
Oxyrrhis marina	MMETSP	Cmb:P0468-71	PEP_0190368498
Paulinella chromatophora	gb		YP_002049510
Physcomitrella patens	gb		XP_001759718.1
*Symbiodinium sp.*CCMP421	MMETSP	P1110	PEP_0181483406
Vitrella brassicaformis	CryptoDB		Vbra_10627, Vbra_16645
